# Efficacy and safety of mesh non-fixation in patients undergoing laparo-endoscopic repair of groin hernia: a systematic review and meta-analysis

**DOI:** 10.1007/s10029-023-02919-4

**Published:** 2023-11-13

**Authors:** F. Kobayashi, J. Watanabe, M. Koizumi, N. Sata

**Affiliations:** 1 Department of Surgery, Division of Gastroenterological, General, and Transplant Surgery, Jichi Medical University, Shimotsuke city, Tochigi Japan; 2 Division of Community and Family Medicine, Jichi Medical University, 3311-1 Yakushiji, Shimotsuke city, Tochigi 329-0498 Japan

**Keywords:** Chronic pain, Hernia recurrence, Laparoscopic inguinal hernia repair, Laparoscopic transabdominal preperitoneal repair, Mesh fixation

## Abstract

**Purpose:**

To examine updated evidence on the efficacy and safety of mesh non-fixation in patients undergoing laparo-endoscopic repair of groin hernias.

**Methods:**

We searched MEDLINE, Cochrane Central Library, Embase, ClinicalTrials. gov, and ICTRP databases to identify randomized controlled trials. The primary outcomes were recurrence, chronic pain, and return to daily life. The certainty of evidence (CoE) was assessed by grading recommendations, assessments, developments, and evaluations. We performed a subgroup analysis based on the surgical type. This study was registered with PROSPERO (CRD 42022368929).

**Results:**

We included 25 trials with 3,668 patients (4,038 hernias) were included. Mesh non-fixation resulted in little to no difference in hernia recurrence (relative risk [RR]:1.40, 95% confidence interval [CI]:0.59–3.31; I^2^ = 0%; moderate CoE) and chronic pain (RR:0.48, 95% CI:0.13–1.78; I^2^ = 77%; moderate CoE), but reduced return to daily life (mean difference [MD]: − 1.79 days, 95% CI: − 2.79 to –0.80; I^2^ = 96%; low CoE). In subgroup analyses, the transabdominal preperitoneal approach (TAPP) (MD: − 2.97 days, 95% CI: − 4.87 to − 1.08; I^2^ = 97%) reduced return to daily life than total extraperitoneal inguinal approach (MD: − 0.24 days, 95% CI − 0.71 to 0.24; I^2^ = 61%) (p = 0.006).

**Conclusions:**

Mesh nonfixation improves the return to daily life without increasing the risk of hernia recurrence or chronic pain. Surgeons and patients may discuss mesh nonfixation options to accommodate a patient’s desired return to daily life. Further trials focusing on TAPP are required to confirm these findings.

**Supplementary Information:**

The online version contains supplementary material available at 10.1007/s10029-023-02919-4.

## Introduction

Groin hernia repair is one of the most frequently performed general surgical procedures, with > 20 million patients undergoing standby repair worldwide [1]. Of all groin hernias, 96% were inguinal, and 4% were femoral [1]. The estimated lifetime risk of groin hernia repair was 27% in men and 3% in women, respectively [2, 3]. Lichtenstein tension-free hernia repair, is the most commonly performed technique with low recurrence and complication rates [4]. With the advent of innovative surgical platforms, minimally invasive approaches such as laparoscopic transabdominal preperitoneal repair (TAPP), total extraperitoneal repair (TEP), and robotic TAPP have emerged [1, 4, 5]. These minimally invasive approaches have similar wound-related complications, recurrence rates, and a more rapid return to work and activity than the Lichtenstein procedure [6, 7]. However, the recovery time and recurrence after groin hernia repair pose major socioeconomic problems, with chronic pain that interferes with daily life and employment occurring in approximately 6% of patients [8].

Mesh non-fixation is considered a means of avoiding the chronic pain associated with fixation devices; however, the high risk of recurrence is a primary concern among surgeons. Although various mesh fixation strategies exist, and a recent network meta-analysis suggested that absorbable tacks and adhesives may minimize recurrence and complications, no statistically or clinically applicable differences were found between strategies. Recent systematic reviews of mesh non-fixation showed that the risk of recurrence, complications, and postoperative pain did not differ between fixation and non-fixation; however, these reviews did not assess the certainty of evidence (CoE) using the Grading of Recommendations, Assessment, Development, and Evaluations (GRADE) approach [9–11]. Therefore, the controversy has not subsided, and more randomized controlled trials (RCTs) have addressed this topic [12–14].

In this study, we aimed to assess the efficacy and safety of mesh nonfixation in patients who underwent groin hernia repair using the GRADE approach.

## Methods

### Protocol

We followed the Preferred Reporting Items for Systematic Reviews and Meta-Analysis (PRISMA) 2020 [15]. The protocol was registered in PROSPERO (CRD 42022368929) and OSF (https://doi.org/10.17605/OSF.IO/RDHGX).

### Study selection

This systematic review included RCTs assessing the efficacy of mesh fixation versus non-fixation and excluded non-RCTs. Adults aged ≥ 18 years who underwent laparo-endoscopic repair of groin hernias were included in this systematic review. Participants who could not tolerate a fixed mesh, such as those with metal allergies, were excluded. The intervention involved mesh non-fixation, which included non-fixation or self-gripping. The control was mesh fixation, including tacks, clips, staples, sutures, glue, and cyanoacrylate, as different types of mesh fixation are considered equally effective [16].

The primary outcomes were hernia recurrence, chronic postoperative inguinal pain (CPIP), and the number of days to return to daily life. Hernia recurrence was defined as the number of recurrent hernias of a single hernia on the side that underwent repair as directly diagnosed by an independent healthcare provider. Hernia formation at previously unrepaired or reinforced sites was excluded. CPIP was defined as bothersome moderate pain affecting daily activities, lasting at least 3 months postoperatively and decreasing over time. Days to return to daily life were defined as the period from surgery date to the return to normal daily activities. Secondary outcomes were postoperative pain, length of hospital stay (days), operative time (min), cost (US$), and adverse events. Postoperative pain was defined as the mean visual analog scale (VAS) or numeric rating scale (NRS) score for pain at rest or during activity on postoperative day (POD) 1. The final score was adopted after pain was measured multiple times. Pain at rest and during activity was measured. We modified the protocol to include cost as a secondary outcome.

We searched the following databases for studies published until October 20, 2022, the Cochrane Central Register of Controlled Trials (CENTRAL) using the Cochrane Library (inception–present), MEDLINE using PubMed (1966–present), and EMBASE using ProQuest Dialog (1988–present) (Supplementary 1). We also searched the World Health Organization International Clinical Trials Platform Search Portal (ICTRP) (inception–present) and ClinicalTrials.gov (inception–present) databases for ongoing and unpublished trials (Supplementary 2). We initially searched for studies published before October 20, 2022, and an updated search on May 2, 2023. We checked the reference lists of all eligible studies, including international guidelines [1, 4], and those citing eligible studies. Studies were not excluded based on the observation period, publication year, language, or country restrictions. All papers included published and unpublished articles, conference abstracts, and letters. We asked the authors of the original studies for unpublished or additional data.

### Data collection and analysis

Two reviewers (FK and JW) independently screened the titles and abstracts, followed by an assessment of eligibility based on the full text. The same two reviewers (FK and JW) performed independent data extraction and evaluated the risk of bias using the risk of bias tool version 2 [17]. Disagreements were resolved through discussion. If a consensus could not be reached, a third reviewer acted as an arbiter to make the final decision (MK).

We pooled the risk ratio (RR) and 95% confidence intervals (CI) for hernia recurrence and CPIP according to the Cochrane Handbook [18], and the mean differences (MD) and 95% Cis for days to return to daily life, length of hospital stay, and operative time. Furthermore, we pooled the effect estimates using standard mean differences (SMDs) for postoperative pain measured using VAS and NRS. We summarized adverse events based on the definitions in the original article; however, no meta-analysis of adverse events was performed. We conducted an intention-to-treat analysis of the dichotomous data. We did not impute missing data for continuous based on the Cochrane Handbook recommendations [18]. We performed a meta-analysis using the data available in the original study and Review Manager software 5.4.2 (Cochrane Collaboration, London, UK) with a random-effects model.

Statistical heterogeneity was evaluated by visually inspecting forest plots and calculating I^2^ (0–40% might not be important; 30–60% may represent moderate heterogeneity; 50–90% may represent substantial heterogeneity; and 75–100%, considerable heterogeneity) based on the Cochrane Handbook [18]. The reasons for were assessed when substantial heterogeneity (I^2^ > 50%) was observed. We performed an extensive literature search of unpublished trials using the Clinical Trial Registry System (ClinicalTrials.gov and ICTRP). Following this, we performed a funnel plot and Egger’s test when ˃10 eligible studies were included in the meta-analysis based on the Cochrane Handbook guidelines [18].

A table summarizing our findings was created based on the Cochrane Handbook [18]. We adopted the corresponding risks from the medians of the included trials. We have included grading to evaluate the CoE based on the GRADE approach for each summary in the findings table [19].

### Additional analyses

To clarify the influence of effect modifiers on the results, we conducted subgroup analyses of the primary outcomes based on the following factors: surgical type (TEP or TAPP), anesthesia type (spinal or general), and fixation type (tack, clip, staple, suture, glue, or cyanoacrylate). However, a subgroup analysis of the anesthesia type regarding recurrence and CPIP could not be performed because of the lack of outcomes in spinal anesthesia trials. We modified the protocol to include a subgroup analysis of the mesh type (polypropylene or three-dimensional (3D)/self-gripping mesh).

We performed the following sensitivity analyses of the primary outcomes to assess the robustness of the review results to decisions made during the review process: exclusion of studies with missing data, exclusion of studies in which hernia recurrence was defined according to the original authors’ definition, and exclusion of studies in which CPIP was defined according to the original authors’ definition. However, we could not perform a sensitivity analysis by excluding studies in which hernia recurrence or CPIP was defined according to the original authors’ definition because there were no such trials. We performed a sensitivity analysis to exclude studies that did not meet the criteria for properly designed trials.

## Results

**Fig. 1:**
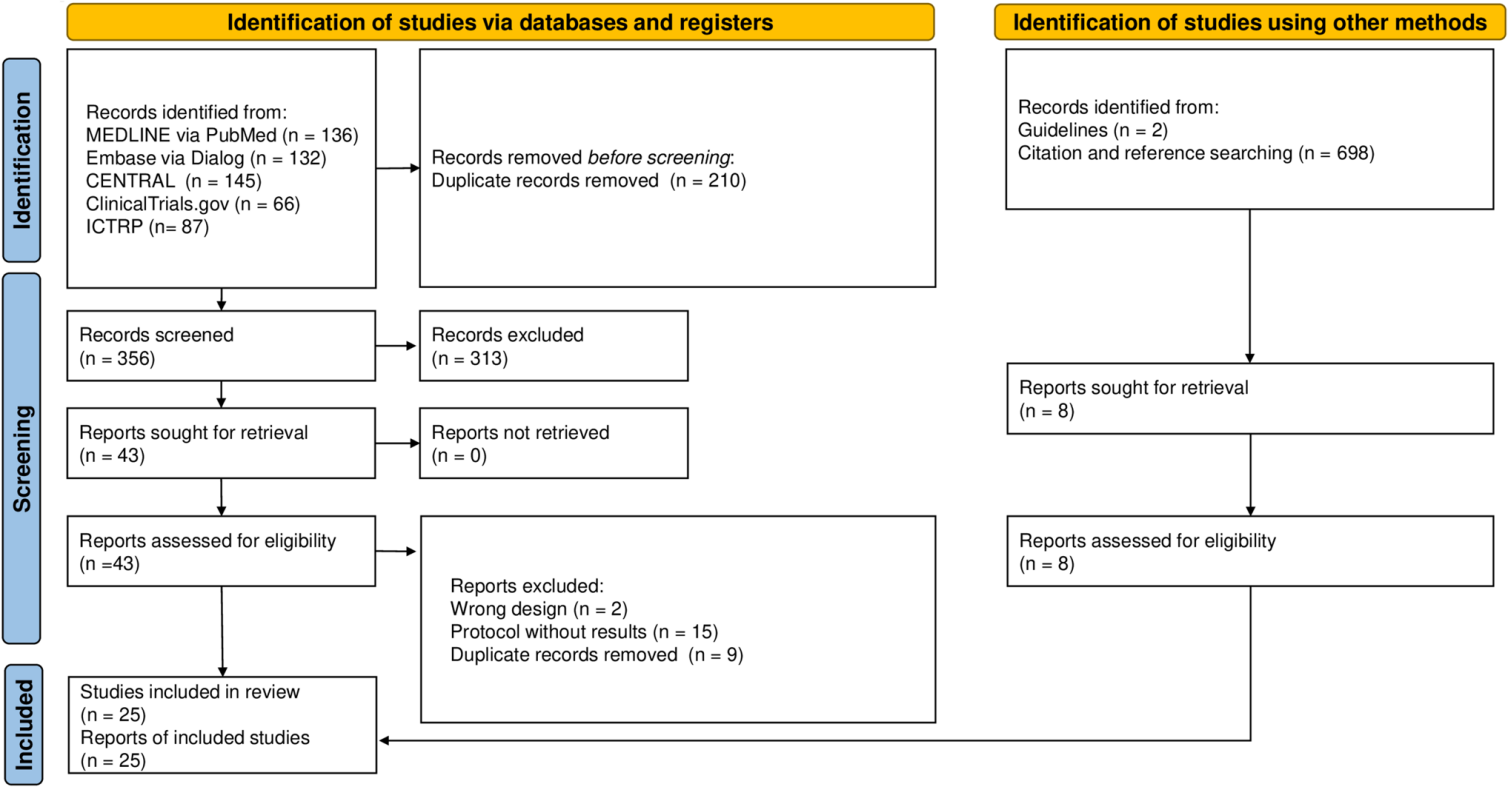
Flow of the study selection process

Figure 1 shows a flowchart of trial selection. After removing the duplicate records, 356 records were identified. After screening, we included 25 trials involving 3,668 patients (4,038 total hernias) [12–14, 20–41].

Table 1 presents the characteristics of the eligible studies. Of the 25 trials, 14 employed TEP approaches, and 11 used TAPP approaches. General anesthesia was administered in 16 trials, spinal anesthesia in one, general or spinal anesthesia in one, and unknown anesthesia in seven. Mesh fixation approaches involved staples in six trials, tacks in 14, sutures in one, glue in one, tacks or glue in two, and tacks and sutures in one. A polypropylene mesh was used in 17 trials, a 3D anatomical mesh in two, and a self-gripping mesh in six.

**Table 1:** Summary of the characteristics of the eligibility studies

Authors [ref no.]	Year	Country	Subject (hernia) no (non-fixation/fixation)	Age (years) (non-fixation/fixation)	Type of hernia according to EHS classification (non-fixation/fixation)	Surgical approach	Mesh of non-fixation	Control (methods of fixation)	Number of fixation or suture	Anesthesia (spinal/general)	Follow-up (months)
Ferzli [20]	1999	USA	49 (50)/ 43 (50)	53/55	L: 33/23, M: 16/20, L + M: 1/7, F: 0/1	TEP	Polypropylene	Staple	4	0/92	12
Smith [21]	1999	UK, Australia	253 (263)/ 249 (273)	53/54	NR	TAPP	Polypropylene	Staple	NR	NR	16^**^
Moreno-Egea [22]	2004	Spain	85/85	57/54	L: 32/23, M: 53/62	TEP	Self-gripping	Staple	NR	41/129	24
Parshad [23]	2005	India	25/25	47/46	NR	TEP	Polypropylene	Staple	NR	NR	23^**^
Koch [24]	2006	USA	20 (27)/ 20 (26)	55/56	L: 12/10, M: 12/13, L + M: 2/3, F: 1/0	TEP	3-dimensional, anatomical mesh	Tacker	5–8	0/40	12
Li [25]	2007	China	30/30	58/61	NR	TAPP	Self-gripping	Staple	NR	NR	NR
Toylor [26]	2008	Australia	180 (250)/ 180 (250)	60/59	(Defect size < 2 cm: 74%) L: 32/31, M: 15/15, L + M: 5/5. F: 2/2, Recurrence 6/6	TEP	Polypropylene	Tacker	7–10^a^	0/360	8.2^b^
Bittner [27]	2011	Germany	150/150	52/53	(Defect size < 3 cm) L: 81/82, M: 43/41, L + M: 5/8, F: 3/0, Recurrence 18/19	TAPP	Polypropylene	Suture	2	0/300	12
Garg [28]	2011	India	52 (96)/ 52 (98)	52/47	NR	TEP	Polypropylene	Tacker	2	104/0	24
Ayyaz [29]	2015	Pakistan	31/32	31/45	L: 25/26, M: 6/6	TEP	Polypropylene	Tacker	2	0/63	60
Claus [30]	2016	Brazil	50/10	51/49	L: 14/2, M: 13/3, L + M: 22/5, F: 1/0	TEP	Polypropylene	Tacker	4–6	0/60	3
Buyukasik [31]	2017	Turkey	50 (68)/ 50 (70)	31/27	L: 30/26, M: 28/30, L + M: 8/12, F: 2/2	TEP	Polypropylene	Tacker	4–7	0/100	12
Li [32]	2017	China	50/50	44/43	NR	TAPP	3-dimensional, anatomical mesh	Staple	NR	0/100	6
Reddy [33]	2017	India	15/15	NR	NR	TEP	NR	Tacker	NR	0/30	1
Shen [34]	2017	China	80/80	60/56	NR	TEP	Polypropylene	Glue	0	0/160	12
Abd-Raboh [35]	2018	Egypt	27 (32)/ 31 (35)	37/36	L: 26/31, M: 4/3, L + M: 2/1	TEP	Polypropylene	Tacker	≥ 3	0/58	6
Lau [36]	2018	Malaysia	40/32	NR	NR	TEP	Self-gripping	Tacker	NR	NR	6
Wang [37]	2018	China	38/38	47/47	NR	TAPP	Polypropylene	Tacker	NR	0/76	NR
Kalidarei [38]	2019	Iran	39/41	54/51	L: 30/35, M: 9/6	TAPP	Polypropylene	Tacker/suture	NR	0/80	6
Khalil [39]	2019	Egypt	15/15	38/40	NR	TAPP	Self-gripping	Tacker	NR	NR	3
Habeeb [12]	2020	Egypt	266/532	NR	NR	TAPP	Polypropylene	Tacker/glue	NR	NR	18
Zayed [40]	2020	Egypt	50/50	43/44	L (≤ 4 cm): 50/50	TAPP	Polypropylene	Tacker	NR	0/100	12
Azevedo [13]	2022	Brazil	21/42	NR	L1: 1, L2: 46, L3: 6, M1: 2, M2: 8, M3: 0	TAPP	NR	Tacker/glue	NR	NR	24
Yildirim [14]	2022	Turkey	50/50	51/52	NR	TEP	Polypropylene	Tacker	NR	0/100	6
Meshkati Yazd [41]	2023	Iran	50/50	40/42	NR	TAPP	Polypropylene	Tacker	2–4	0/100	6

*EHS* European hernia society, *F* femoral, *L* lateral, *M* medial, *NR* not reported, *TAPP* Transabdominal preperitoneal approach, *TEP* total extraperitoneal inguinal approach

^a^Main number

^b^Median times

Table 2 displays the risk of bias for recurrence, with 21 trials exhibiting ‘some concerns’ and two trials presenting a ‘high’ risk of bias.

**Table 2:** Risk of bias for the eligibility studies for patients’ pains

Authors [ref no.]	Risk of bias 2 assessment					
	Bias arising from the randomization process	Bias due to deviations from intended interventions	Bias due to missing outcome data	Bias in measurement of the outcome	Bias in selection of the reported results	Overall risk of bias
Ferzli [20]	Some concerns	Low	Low	Some concerns	Some concerns	Some concerns
Smith [21]	Some concerns	Low	Some concerns	Some concerns	Some concerns	Some concerns
Moreno-Egea [22]	Some concerns	Low	Low	Low	Some concerns	Some concerns
Parshad [23]	Low	Low	Some concerns	Some concerns	Some concerns	Some concerns
Koch [24]	Some concerns	Low	Some concerns	Some concerns	Some concerns	Some concerns
Li [25]	Low	Low	Some concerns	Some concerns	Some concerns	Some concerns
Tolver [26]	Low	Low	Some concerns	Low	Some concerns	Some concerns
Bittner [27]	Low	Low	Low	Some concerns	Some concerns	Some concerns
Garg [28]	High	Low	Some concerns	Low	Some concerns	High
Ayyaz [29]	High	Low	Low	Low	Some concerns	High
Claus [30]	Some concerns	Low	Low	Some concerns	Some concerns	Some concerns
Buyukasik [31]	Low	Low	Some concerns	Some concerns	Some concerns	Some concerns
Li [32]	Low	Low	Low	Low	Some concerns	Some concerns
Shen [34]	Some concerns	Low	Low	Some concerns	Some concerns	Some concerns
Abd-Raboh [35]	Some concerns	Low	Low	Some concerns	Some concerns	Some concerns
Wang [37]	Some concerns	Low	Some concerns	Low	Low	Some concerns
Kalidarei [38]	Low	Low	Some concerns	Some concerns	Some concerns	Some concerns
Khalil [39]	Low	Low	Low	Some concerns	Some concerns	Some concerns
Habeeb [12]	Low	Low	Low	Some concerns	Some concerns	Some concerns
Zayed [40]	Some concerns	Low	Some concerns	Some concerns	Some concerns	Some concerns
Azevedo [13]	Low	Low	Some concerns	Some concerns	Some concerns	Some concerns
Yildirim [14]	Low	Low	Low	Low	Some concerns	Some concerns
Meshkati Yazd [41]	Low	Low	Low	Some concerns	Low	Some concerns

The risk of bias using Risk of Bias 2; Low: Risk of bias was low. Some concerns, Risk of bias was some concerns. High; Risk of bias was high

### Outcomes

Table 3 summarizes the findings of the GRADE approach.

**Table 3:** Summary of findings

The efficacy and safety of non-fixation of mesh in patients undergoing laparoscopic inguinal hernia repair						
Patient or population: adults, setting: herniorrhaphy, intervention: non-fixation, comparison: fixation						
Outcomes	Anticipated absolute effects* (95% CI)		Relative effect (95% CI)	Patient number (Studies)	Certainty of the evidence (GRADE)	Comments
	Risk with fixation	Risk with non-fixation				
Recurrence	5 per 1,000	7 per 1,000 (3 to 16)	RR 1.40 (0.59 to 3.31)	3,796 (23 RCTs)	Moderate^a^	Mesh non-fixation likely results in little to no difference in hernia recurrence
Chronic postoperative inguinal hernia pain	85 per 1,000	41 per 1,000 (11 to 151)	RR 0.48 (0.13 to 1.78)	1,713 (9 RCTs)	Moderate^a^	Mesh non-fixation likely results in little to no difference in CPIP
Return to daily life (days)	The median return to daily life was 8 days	MD -1.58 days (− 2.46 to − 0.69)	–	1,122 (9 RCTs)	Low^b^	Mesh non-fixation may reduce return to daily life
Postoperative pain on day 1	–	SMD -0.53 days (-1.00 to 0.06)	–	1,081 (12 RCT)	Low^b^	Mesh non-fixation may reduce postoperative pain on day 1
Length of hospital stay (days)	The median length of hospital stay was 1.67 day	MD 0.72 days (− 2.10 to 0.66)	–	1,078 (13 RCTs)	Low^b^	Mesh non-fixation may result in little to no difference in length of hospital stay
Operative time (minutes)	The median operative time was 47.5 min	MD 2.17 min (− 3.80 to − 0.53)	–	2,100 (4 RCTs)	Low^b^	Mesh non-fixation may result in little to no difference in operative time

*The risk in the intervention group (and its 95% CI) was based on the assumed risk in the comparison group and the relative effect of the intervention (and its 95% CI). GRADE Working Group grades of evidence: High certainty. We are confident that the true effect is close to the estimated effect. Moderate certainty: We are moderately confident about the estimated effects. The true effect is likely close to the estimated effect; however, it may be substantially different. Low certainty: Confidence in the estimated effect is limited, and the true effect may differ substantially from the estimated effect. Very low certainty: We have very little confidence in the estimated effects. The true effect is likely to differ substantially from the estimated effect.

*CI* confidence interval, *CPIP* chronic postoperative inguinal hernia pain, *MD* mean difference, *RR* risk ratio, *SMD* standard mean difference.

^a^Downgraded points because of imprecision due to a wide interval or small sample size. ^b^Two points were downgraded because of inconsistencies due to substantial heterogeneity

### Primary outcomes

**Fig. 2:**
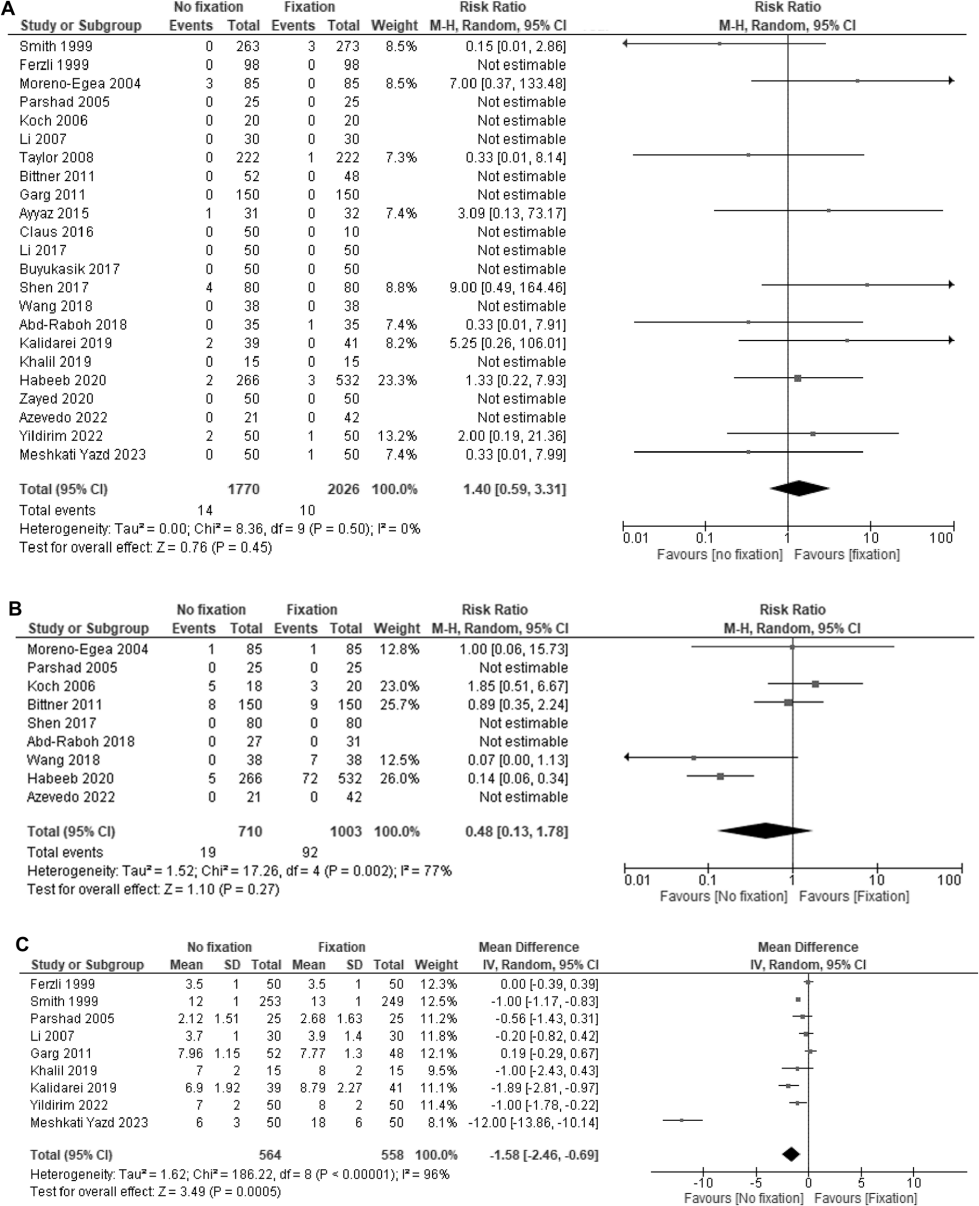
Forest plot **A** recurrence; **B** chronic postoperative inguinal pain; **C** return to daily life

Twenty-three trials reported recurrence [12–14, 20–32, 34, 35, 37–41]. Of these, 13 patients did not experience recurrence. Mesh non-fixation likely resulted in little to no difference in hernia recurrence compared to mesh fixation (RR: 1.40, 95% CI: 0.59–3.31; I^2^ = 0%; moderate CoE; Fig. 2A).

Nine trials reported on CPIP [12, 13, 22–24, 27, 34, 35, 37]. Of these, four trials did not report CPIP [13, 23, 34, 35], and five trials did not report the number of tacks [12, 13, 22, 23, 37]. Mesh non-fixation resulted in little to no difference in CPIP compared with mesh fixation (RR: 0.48, 95% CI: 0.13 to 1.78; I^2^ = 77%; moderate CoE; Fig. 2B).

Nine trials reported return to daily life [14, 20, 21, 23, 25, 28, 38, 39, 41]. The median return to daily life with mesh fixation was 8 days. Mesh non-fixation may improve return to daily life than mesh fixation (MD: − 1.79 days, 95% CI: − 2.79 to − 0.80; I^2^ = 96%; low CoE; Fig. 2C).

### Secondary outcomes

**Fig. 3:**
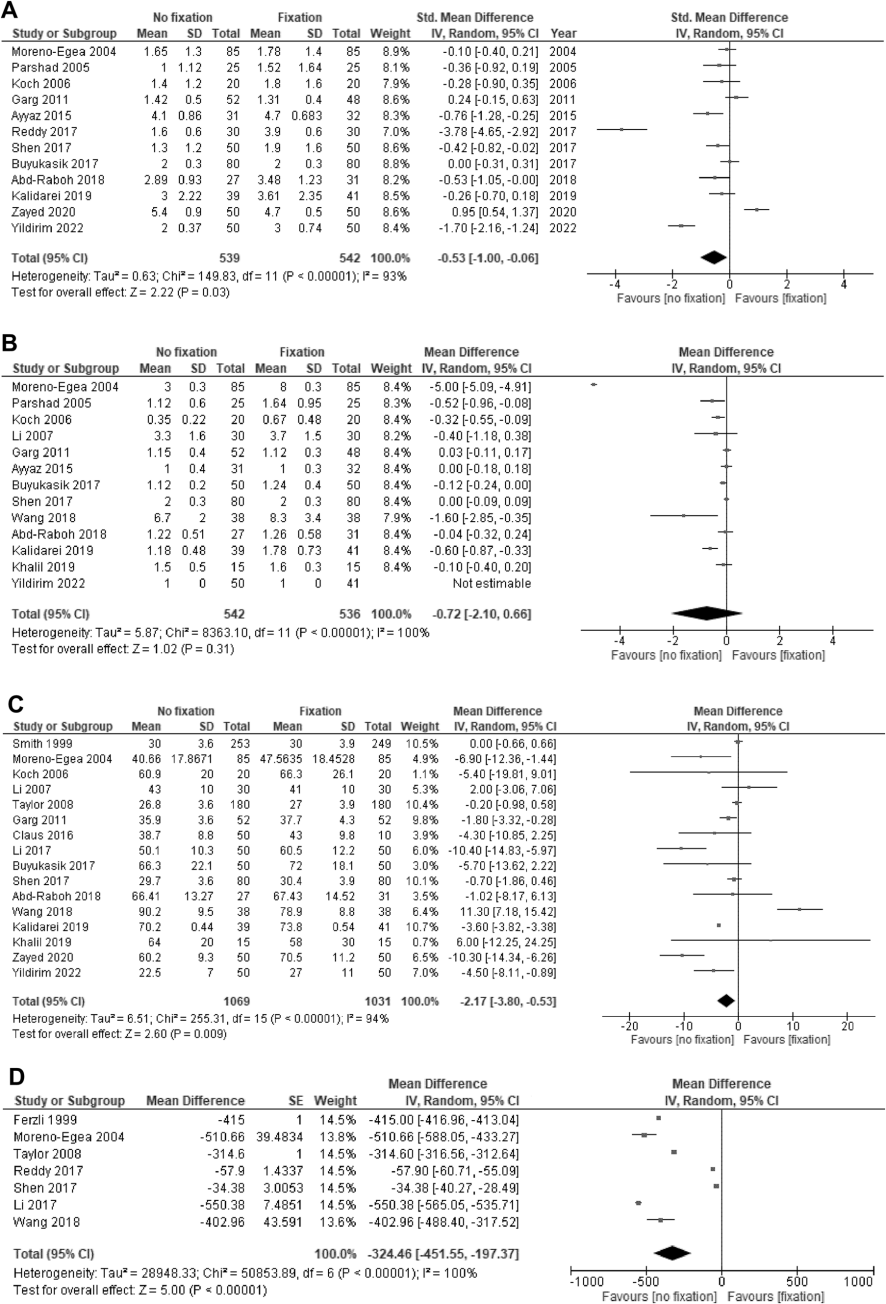
Forest plot **A** pain on postoperative day 1; **B** length of hospital stay; **C** operative time; **D** Cost

Twelve trials reported pain on POD 1 [14, 22–24, 28, 29, 31, 33–35, 38, 40]. Of those, nine used the VAS [14, 22, 23, 29, 33–35, 38, 40], and three used the NRS [24, 28, 31]. Mesh non-fixation may reduce pain on POD 1 compared with mesh fixation (SMD: − 0.53 days, 95% CI: − 1.00 to − 0.06; I^2^ = 93%; low CoE; Fig. 3A).

Thirteen trials reported the length of hospital stay [14, 22–25, 28, 29, 31, 34, 35, 37–39]. Mesh non-fixation may result in little to no difference in the length of hospital stay compared with mesh fixation (MD: − 0.72 days, 95% CI: − 2.10 to 0.66; I^2^ = 100%; low CoE; Fig. 3B).

Sixteen trials reported operative time [14, 21, 22, 24–26, 28, 30–32, 34, 35, 37–40]. Mesh non-fixation may reduce operative time compared with mesh fixation (MD: -2.17 min, 95% CI: − 3.80 to − 0.53; I^2^ = 94%; low CoE; Fig. 3C).

Seven trials reported costs [20, 22, 26, 32–34, 37]. Mesh non-fixation may reduce costs compared with mesh fixation (MD: –324.5 US$, 95% CI: − 451.6 to − 197.4; I^2^ = 100%; low CoE; Fig. 3D).

Twenty-three trials reported adverse events [12–14, 20–24, 26–32, 34–41]. Seroma, hematoma, and urinary retention were the primary complications. Seroma and hematoma are similar with and without fixation [12–14, 20, 21, 23, 24, 27–32, 34, 35, 37, 38, 40], whereas mesh non-fixation tends to reduce urinary retention [21, 24, 28, 29, 31, 38].

### Additional analyses

We performed subgroup and sensitivity analyses (Supplementary Figs. 1–4). In the subgroup analysis of fixation type, mesh non-fixation reduced CPIP than mesh fixation with glue (RR: 0.14, 95% CI: 0.06–0.34), while mesh non-fixation resulted in little to no difference in CPIP compared with mesh fixation with tacker, staple, or suture (test for subgroup differences, p = 0.004). Mesh non-fixation with TAPP (MD: − 3.84 days, 95% CI: − 6.88 to − 0.80; I^2^ = 98%) improved the return to daily life than that with TEP (MD: − 0.24, 95% CI:− 0.71 to 0.24; I^2^ = 61%) (test for subgroup differences, p = 0.02). In the subgroup analysis of anesthesia type, mesh non-fixation with general anesthesia (MD: − 3.03 days, 95% CI: − 5.48 to − 0.58; I^2^ = 98%) improved return to daily life than that spinal anesthesia (MD: 0.19 days, 95% CI: − 0.29 to 0.67) (test for subgroup differences, p = 0.02). In the subgroup analysis of mesh type, non-fixation with polypropylene mesh (MD: − 1.90 days, 95% CI: − 2.97 to − 0.83; I^2^ = 97%) improved return to daily life faster than that with 3D or self-gripping mesh (MD: − 0.33 days, 95% CI: − 0.90 to 0.25; I^2^ = 1%) (test for subgroup differences, p = 0.01). The other subgroup analyses did not differ significantly (test for subgroup differences, p ≥ 0.05). After excluding studies with missing data [13, 21, 23–26, 28, 31, 37, 38, 40] and those that did not meet the criteria for a properly designed trial [13, 29, 39, 40], sensitivity analyses were consistent with the main results (Supplementary 3). Publication bias was not detected as the funnel plot was symmetrical (Egger’s test, p = 0.785; Supplementary Fig. 5).

## Discussion

This systematic review and meta-analysis, including 25 trials with 3,668 patients (4,038 total hernias), demonstrated that mesh non-fixation improved return to daily life without increasing recurrence. However, little to no difference in CPIP was observed. Additionally, it reduced pain on POD 1, operative time, and costs without increasing the incidence of adverse events. This study provides new evidence that mesh non-fixation may improve the return to daily activities; therefore, it is important for surgeons and patients.

Our finding that mesh non-fixation did not increase hernia recurrence is consistent with previous findings [9–11]. Up to 2% of hernia recurrence after minimally invasive repair has been reported for TEP and TAPP repairs [1, 6]. Various risk factors for hernia recurrence have been identified, including mesh fixation (tack, clip, staple, suture, or glue), anesthesia type, mesh type, medial or lateral hernia sac, sliding hernia, operative time, registry database participation, femoral hernia, proper dissection and space creation, postoperative complications, and center/surgeon volume [1, 42]. The present meta-analysis included only RCTs; subgroup analyses of anesthesia and fixation type did not reveal any differences. Furthermore, the absence of heterogeneity (I^2^ = 0%) indicated less variability across studies, providing consistency and reliability of our findings.

The inclusion of a standard polypropylene mesh (> 50 g/m^2^) and the exclusion of large hernias, especially M3 hernias, according to the European Hernia Society classification, may limit the generalizability of the findings in this study. Notably, the 2018 international guidelines of the HerniaSurge Group recommended mesh fixation in M3 hernias (large medial) to reduce the risk of recurrence in both TEP and TAPP [1]. However, recent experimental and large database studies have suggested that mesh fixation may be unnecessary [43, 44]. Moreover, a recent meta-analysis and large database study reported that using a lightweight mesh (≤ 50 g/m^2^) in the laparo-endoscopic repair of direct or large inguinal hernias can potentially increase recurrence rates [44, 45]. A heavy mesh (> 70 g/m^2^) should be used in cases necessitating direct laparo-endoscopic repair of large inguinal hernias [45]. Another important point is that the International Endohernia Society’s guidelines recommend a mesh size of at least 10 × 15 cm^2^, but for large medial hernias, a larger mesh (i.e. 12 × 17 cm^2^ or larger) is recommended, independent of the fixation [46]. These are ongoing research areas that will be investigated further in future studies.

By conducting a meta-analysis of twice as many studies and cases as previous reviews [9–11], we found that mesh nonfixation improved the return to daily life, particularly in TAPP under general anesthesia. In contrast, previous systematic reviews focusing on TEP [9–11] found that mesh non-fixation did not improve return to daily life. The difference in results can be attributed to the present subgroup analysis, which found that TAPP improved the return to daily life compared with TEP. The exact mechanism is unknown; however, a reduction of approximately 1.8 days is critical, considering that the return to daily life did not differ between TEP and TAPP in a previous review [47]. Additionally, we found that mesh nonfixation led to a faster return to daily life with general anesthesia than with lumbar anesthesia. With the increasing incidence of groin hernias in the older population, these findings are clinically relevant, especially considering the recommended use of general anesthesia for patients aged ≥ 65 years [1]. This suggests that mesh nonfixation combined with general anesthesia may provide an optimal approach for groin hernia repair in the older population. However, our finding that nonfixation with a polypropylene mesh improved the return to daily life faster than with 3D or self-gripping meshes was inconsistent with that of a previous review. In the Meshkati Yazd study, return to daily life in the fixation group occurred only at 3 weeks postoperatively, and the results were heavily influenced by those of the mesh-type subgroup [41]. Future trials focusing on TAPP under general anesthesia with a preliminary definition of return to daily life are required to confirm these results.

Consistent with the findings of previous studies, mesh non-fixation did not reduce CPIP [10, 16]. This suggests that the mesh fixation method may not significantly influence the development of chronic pain after groin hernia repair. Therefore, other factors or surgical techniques should be considered to address this issue. One study found a significant correlation between the number of tacks used and the incidence of pain, with six tacks being the cutoff point [26]. In this review, subgroup analysis based on the number of tacks was impossible because many studies did not report this number; however, it may be related to the incidence of pain. Surgery is the main cause of postoperative pain after groin hernia repair due to pubic groin, iliopsoas muscle, or popliteal nerve injuries [48]. Therefore, a multifaceted surgical approach is required to prevent the development of CPIPs.

Our findings concerning postoperative pain, length of hospital stay, operative time, and cost were consistent with those of previous reviews [9–11], suggesting that our analysis aligns with prior research in terms of these outcomes and further supports the validity of our results. Moreover, we expand these findings using the GRADE approach to demonstrate low-to-moderate CoEs and provide a more comprehensive understanding of the outcomes.

This study has several limitations. First, although this review included only RCTs, the CoE remained moderate. This may have been due to the limited power and high heterogeneity in some trials. The imprecision and inconsistency of the results may be further refined by adjusting for preoperative patient characteristics (e.g., sex) and hernia characteristics (e.g., primary or bilateral). Second, several confounding factors may have influenced the results, including surgeon experience and expertise, inclusion and exclusion criteria, learning curve, hospital volume, surgical technique, mesh type, fixation technique, outcome reporting, and follow-up duration. These factors should be considered when interpreting the findings of this study. Further studies are required to better understand their impacts on the outcomes of interest.

In conclusion, this systematic review and meta-analysis showed that mesh non-fixation improved the return to daily life without increasing hernia recurrence or chronic pain. Surgeons and patients may discuss the mesh nonfixation option to accommodate the timing of the patient’s desired return to daily life. Further trials focusing on TAPP are required to confirm these findings.

### Supplementary Information

Below is the link to the electronic supplementary material.

Supplementary file1 (PDF 72 KB)

Supplementary file2 (PDF 280 KB)

## Data Availability

All data are available from public databases.
