# Supplementary material for: Efficacy and safety of mesh non-fixation in patients undergoing laparo-endoscopic repair of groin hernia: a systematic review and meta-analysis
Source: Hernia. 2023 Nov 13;27(6):1415–27. doi: 10.1007/s10029-023-02919-4 (PMC10700198; doi:10.1007/s10029-023-02919-4)
Supplement: Supplementary file 1 — Supplementary file1 (PDF 72 KB) [file 10029_2023_2919_MOESM1_ESM.pdf]

Supplementary 1: The electronic database search strategy

CENTRAL search strategy

#1. ([mh "Hernia, Inguinal"] OR [mh "Hernia, Femoral"])

#2. ((inguina *:ti,ab OR groin*:ti,ab OR femoral:ti,ab) AND herni*:ti,ab).

#3. #1 OR #2

#4. (Laparoscopy *ti,ab OR endoscopy *ti,ab OR video*ti,ab OR tap:ti,ab OR transabdominal:ti,ab OR tep:ti,ab OR (total*ti,ab AND extraperitoneal:ti,ab)).

#5. #3 AND #4

#6. (Fixation:ti,ab OR non-fixation:ti,ab OR no-fixation:ti,ab OR self-gripping:ti,ab OR Progrip:ti,ab).

#7. #5 AND #6

MEDLINE (via PubMed)

#1. "Hernia, Inguinal"[Mesh] OR "Hernia, Femoral"[Mesh]

#2. (inguina *[tiab] OR groin*[tiab] OR femoral[tiab]) AND herni*[tiab]

#3. #1 OR #2

#4. (Laparoscopy *[tiab] OR endoscopy *[tiab] OR video*[tiab] OR tap[tiab] OR transabdominal[tiab] OR tep[tiab]) OR (total*[tiab] AND extraperitoneal[tiab])

#5. #3 AND #4

#6. (fixation[tiab] OR nonfixation [tiab] OR no-fixation[tiab] OR self-gripping[tiab] OR progression[tiab])

#7. #5 AND #6

#8. (randomized controlled trial [pt] OR controlled clinical trial [pt] OR randomized [tiab] OR drug therapy[sh] OR placebo [tiab] OR random [tiab] OR trial[tiab] OR

groups[tiab]) NOT (animals [mh] NOT humans [mh]).

#9. #7 AND #8

EMBASE (via ProQuest Dialog) search strategy.

S1 EMB.EXACT.EXPLODE("inguinal hernia")

S2 EMB.EXACT.EXPLODE("femoral hernia")

S3 ab(Inguinal OR groin OR femoral) OR ti(Inguinal OR groin OR femoral).

S4 ab(hernia) OR ti(hernia)

S5 S3 AND S4

S6 S1 OR S2 OR S5

S7 ab(laparoscopic OR laparoscopy OR endoscopic OR endoscopy OR video OR tap OR transabdominal OR tep) OR ti(laparoscopic OR laparoscopy OR endoscopic OR endoscopy OR video OR tap OR transabdominal OR tep).

S8 ab(total AND extraperitoneal) OR ti(total AND extraperitoneal)

S9 S7 OR S8

S10 S6 AND S9

S11: ab(fixation OR non-fixation OR no-fixation OR self-gripping OR Progrip) OR ti(fixation OR non-fixation OR no-fixation OR self-gripping OR Progrip).

S12 S10 AND S11

S13 (ab(random*) OR ti(random*)) OR (ab(placebo*) OR ti(placebo*)) OR (ab(double NEAR/1 blind*) OR ti(double NEAR/1 blind*)).

S14 S12 AND S13

Supplementary 2: The trial registry search strategy

ICTRP search strategy

((Inguinal OR groin OR femoral) AND hernia) AND (fixation OR non-fixation OR non-fixation OR self-gripping OR Progrip)

ClinicalTrials.gov search strategy.

Conditions or diseases: ((Inguinal OR groin OR femoral) AND hernia)

Intervention: (fixation OR non-fixation OR non-fixation OR self-gripping OR Progrip)

Supplementary 3: The results of sensitivity analysis

Sensitivity analysis	Outcomes	No. of studies	Risk Ratio	95% confidence interval
Exclusion of studies with missing data	Recurrence	11	1.78	0.66 to 4.79
	Chronic postoperative inguinal hernia pain	5	0.43	0.09 to 2.06
	Return to daily life (days)	4	–3.37	–6.58 to –0.16
Exclusion of studies which did not meet the criteria of a properly designed trial	Recurrence	19	1.31	0.53 to 3.23
	Chronic postoperative inguinal hernia pain	8	0.48	0.13 to 1.78
	Return to daily life (days)	8	–1.65	–2.59 to –0.70
