# Supplementary material for: Efficacy and safety of mesh non-fixation in patients undergoing laparo-endoscopic repair of groin hernia: a systematic review and meta-analysis
Source: Hernia. 2023 Nov 13;27(6):1415–27. doi: 10.1007/s10029-023-02919-4 (PMC10700198; doi:10.1007/s10029-023-02919-4)

Supplementary Fig. 1A

Study or Subgroup	No fixation Events	No fixation Total	Fixation Events	Fixation Total	Weight	Risk Ratio M-H, Random, 95% CI
**3.1.1 TEP**						
Ferzli 1999	0	98	0	98		Not estimable
Moreno-Egea 2004	3	85	0	85	16.2%	7.00 [0.37, 133.48]
Parshad 2005	0	25	0	25		Not estimable
Koch 2006	0	20	0	20		Not estimable
Taylor 2008	0	222	1	222	13.8%	0.33 [0.01, 8.14]
Garg 2011	0	150	0	150		Not estimable
Ayyaz 2015	1	31	0	32	14.1%	3.09 [0.13, 73.17]
Claus 2016	0	50	0	10		Not estimable
Buyukasik 2017	0	50	0	50		Not estimable
Shen 2017	4	80	0	80	16.7%	9.00 [0.49, 164.46]
Abd-Raboh 2018	0	35	1	35	14.1%	0.33 [0.01, 7.91]
Yildirim 2022	2	50	1	50	25.1%	2.00 [0.19, 21.36]
**Subtotal (95% CI)**		**896**		**857**	**100.0%**	**2.03 [0.62, 6.67]**
Total events	10		3			
Heterogeneity: Tau² = 0.00; Chi² = 4.27, df = 5 (P = 0.51); I² = 0%						
Test for overall effect: Z = 1.17 (P = 0.24)						
**3.1.2 TAPP**						
Smith 1999	0	263	3	273	19.0%	0.15 [0.01, 2.86]
Li 2007	0	30	0	30		Not estimable
Bittner 2011	0	52	0	48		Not estimable
Li 2017	0	50	0	50		Not estimable
Wang 2018	0	38	0	38		Not estimable
Kalidarei 2019	2	39	0	41	18.5%	5.25 [0.26, 106.01]
Khalil 2019	0	15	0	15		Not estimable
Habeeb 2020	2	266	3	532	45.8%	1.33 [0.22, 7.93]
Zayed 2020	0	50	0	50		Not estimable
Azevedo 2022	0	21	0	42		Not estimable
Meshkati Yazd 2023	0	50	1	50	16.7%	0.33 [0.01, 7.99]
**Subtotal (95% CI)**		**874**		**1169**	**100.0%**	**0.90 [0.23, 3.45]**
Total events	4		7			
Heterogeneity: Tau² = 0.20; Chi² = 3.33, df = 3 (P = 0.34); I² = 10%						
Test for overall effect: Z = 0.16 (P = 0.88)						

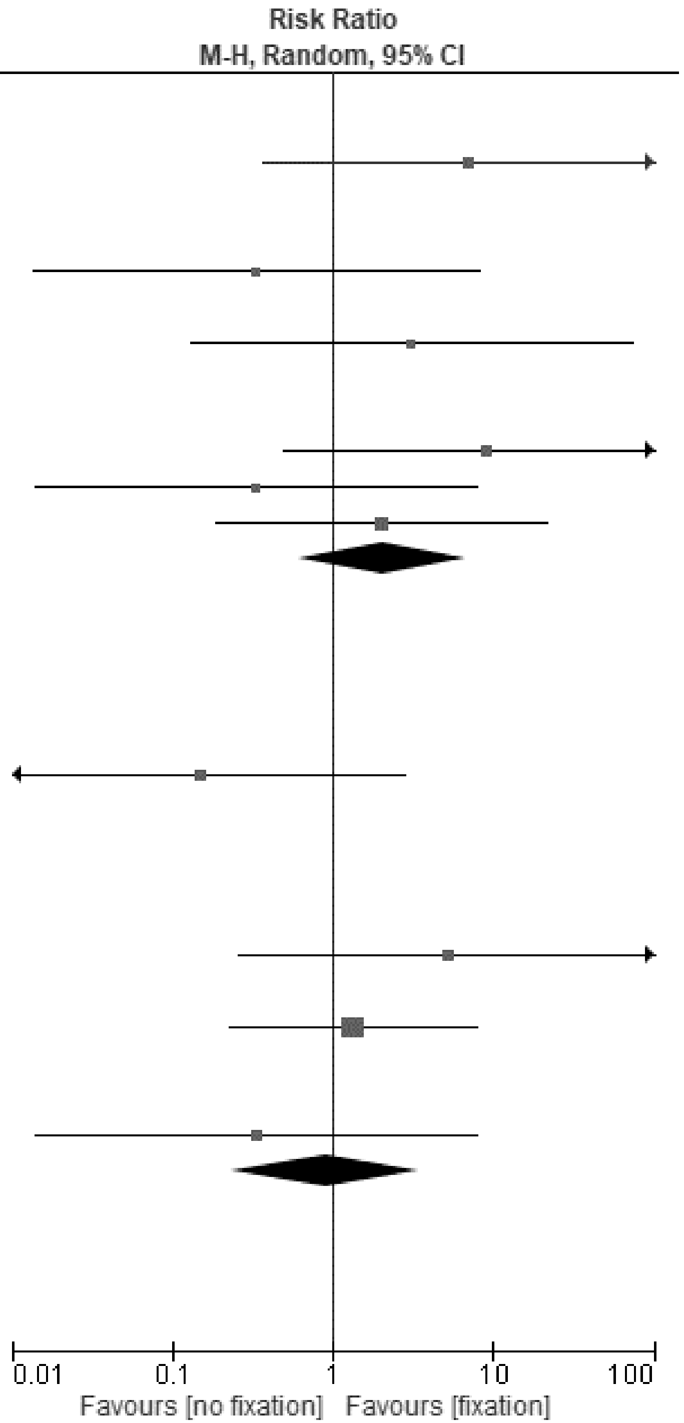

Test for subgroup differences: Chi² = 0.80, df = 1 (P = 0.37), I² = 0%

Supplementary Fig. 1B

Study or Subgroup	No fixation Events	No fixation Total	Fixation Events	Fixation Total	Weight	Risk Ratio M-H, Random, 95% CI
**3.2.1 Tacker**						
Smith 1999	0	263	3	273	11.5%	0.15 [0.01, 2.86]
Ferzli 1999	0	98	0	98		Not estimable
Moreno-Egea 2004	3	85	0	85	11.6%	7.00 [0.37, 133.48]
Parshad 2005	0	25	0	25		Not estimable
Li 2007	0	30	0	30		Not estimable
Garg 2011	0	150	0	150		Not estimable
Ayyaz 2015	1	31	0	32	10.1%	3.09 [0.13, 73.17]
Claus 2016	0	50	0	10		Not estimable
Buyukasik 2017	0	50	0	50		Not estimable
Abd-Raboh 2018	0	35	1	35	10.1%	0.33 [0.01, 7.91]
Wang 2018	0	38	0	38		Not estimable
Kalidarei 2019	2	39	0	41	11.2%	5.25 [0.26, 106.01]
Khalil 2019	0	15	0	15		Not estimable
Zayed 2020	0	50	0	50		Not estimable
Habeeb 2020a	2	266	1	266	17.6%	2.00 [0.18, 21.92]
Yildirim 2022	2	50	1	50	18.0%	2.00 [0.19, 21.36]
Azevedo 2022a	0	21	0	21		Not estimable
Meshkati Yazd 2023	0	50	1	50	10.0%	0.33 [0.01, 7.99]
**Subtotal (95% CI)**		**1346**		**1319**	**100.0%**	**1.39 [0.51, 3.80]**
Total events	10		7			
Heterogeneity: Tau² = 0.00; Chi² = 6.10, df = 7 (P = 0.53); I² = 0%						
Test for overall effect: Z = 0.65 (P = 0.52)						
**3.2.2 Staple**						
Koch 2006	0	20	0	20		Not estimable
Taylor 2008	0	222	1	222	100.0%	0.33 [0.01, 8.14]
Li 2017	0	50	0	50		Not estimable
**Subtotal (95% CI)**		**292**		**292**	**100.0%**	**0.33 [0.01, 8.14]**
Total events	0		1			
Heterogeneity: Not applicable						
Test for overall effect: Z = 0.67 (P = 0.50)						
**3.2.3 Glue**						
Shen 2017	4	80	0	80	38.4%	9.00 [0.49, 164.46]
Habeeb 2020b	2	266	2	266	61.6%	1.00 [0.14, 7.05]
Azevedo 2022b	0	21	0	21		Not estimable
**Subtotal (95% CI)**		**367**		**367**	**100.0%**	**2.32 [0.27, 20.29]**
Total events	6		2			
Heterogeneity: Tau² = 0.99; Chi² = 1.62, df = 1 (P = 0.20); I² = 38%						
Test for overall effect: Z = 0.76 (P = 0.45)						
**3.2.4 Suture**						
Bittner 2011	0	52	0	48		Not estimable
**Subtotal (95% CI)**		**52**		**48**		**Not estimable**
Total events	0		0			
Heterogeneity: Not applicable						
Test for overall effect: Not applicable						

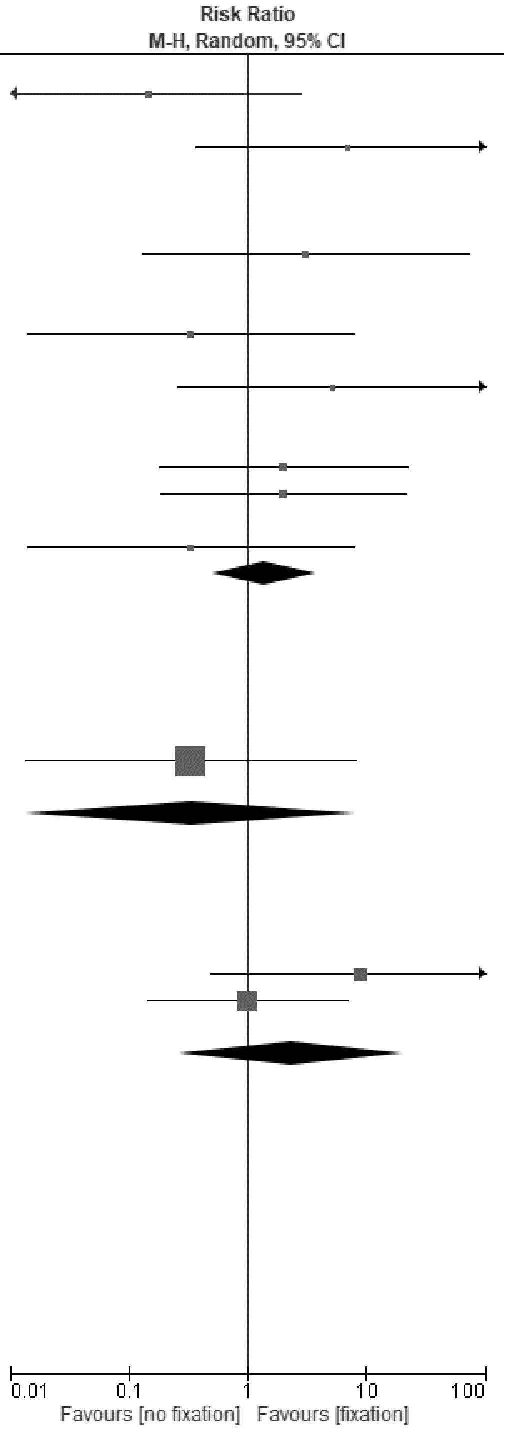

Test for subgroup differences: Chi² = 0.98, df = 2 (P = 0.61), I² = 0%

Supplementary Fig. 1C

Study or Subgroup	No fixation Events	No fixation Total	Fixation Events	Fixation Total	Weight	Risk Ratio M-H, Random, 95% CI
**3.3.1 Polypropylene**						
Smith 1999	0	263	3	273	9.3%	0.15 [0.01, 2.86]
Ferzli 1999	0	98	0	98		Not estimable
Parshad 2005	0	25	0	25		Not estimable
Taylor 2008	0	222	1	222	7.9%	0.33 [0.01, 8.14]
Bittner 2011	0	52	0	48		Not estimable
Garg 2011	0	150	0	150		Not estimable
Ayyaz 2015	1	31	0	32	8.1%	3.09 [0.13, 73.17]
Claus 2016	0	50	0	10		Not estimable
Buyukasik 2017	0	50	0	50		Not estimable
Shen 2017	4	80	0	80	9.6%	9.00 [0.49, 164.46]
Wang 2018	0	38	0	38		Not estimable
Abd-Raboh 2018	0	35	1	35	8.1%	0.33 [0.01, 7.91]
Kalidarei 2019	2	39	0	41	9.0%	5.25 [0.26, 106.01]
Habeeb 2020	2	266	3	532	25.5%	1.33 [0.22, 7.93]
Zayed 2020	0	50	0	50		Not estimable
Yildirim 2022	2	50	1	50	14.5%	2.00 [0.19, 21.36]
Meshkati Yazd 2023	0	50	1	50	8.0%	0.33 [0.01, 7.99]
**Subtotal (95% CI)**		**1549**		**1784**	**100.0%**	**1.20 [0.49, 2.96]**
Total events	11		10			
Heterogeneity: Tau² = 0.00; Chi² = 7.10, df = 8 (P = 0.53); I² = 0%						
Test for overall effect: Z = 0.40 (P = 0.69)						
**3.3.2 3D or self-gripping**						
Moreno-Egea 2004	3	85	0	85	100.0%	7.00 [0.37, 133.48]
Koch 2006	0	20	0	20		Not estimable
Li 2007	0	30	0	30		Not estimable
Li 2017	0	50	0	50		Not estimable
Khalil 2019	0	15	0	15		Not estimable
**Subtotal (95% CI)**		**200**		**200**	**100.0%**	**7.00 [0.37, 133.48]**
Total events	3		0			
Heterogeneity: Not applicable						
Test for overall effect: Z = 1.29 (P = 0.20)						

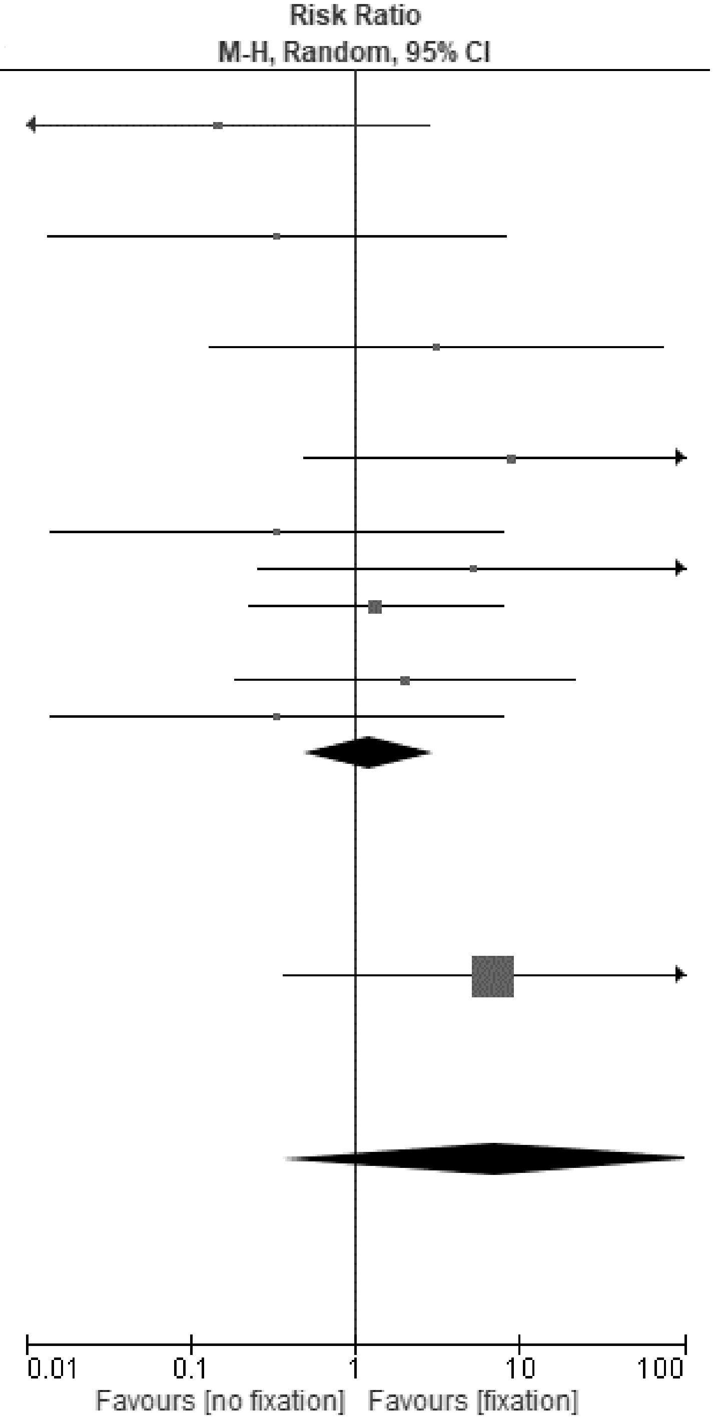

Test for subgroup differences: Chi² = 1.26, df = 1 (P = 0.26), I² = 20.3%

Supplementary Fig. 2A

Study or Subgroup	No fixation Events	No fixation Total	Fixation Events	Fixation Total	Weight	Risk Ratio M-H, Random, 95% CI
**3.4.1 TEP**						
Moreno-Egea 2004	1	85	1	85	17.8%	1.00 [0.06, 15.73]
Parshad 2005	0	25	0	25		Not estimable
Koch 2006	5	18	3	20	82.2%	1.85 [0.51, 6.67]
Shen 2017	0	80	0	80		Not estimable
Abd-Raboh 2018	0	27	0	31		Not estimable
**Subtotal (95% CI)**		**235**		**241**	**100.0%**	**1.66 [0.52, 5.31]**
Total events	6		4			
Heterogeneity: Tau² = 0.00; Chi² = 0.16, df = 1 (P = 0.69); I² = 0%						
Test for overall effect: Z = 0.85 (P = 0.39)						
**3.4.2 TAPP**						
Bittner 2011	8	150	9	150	40.2%	0.89 [0.35, 2.24]
Wang 2018	0	38	7	38	19.4%	0.07 [0.00, 1.13]
Habeeb 2020	5	266	72	532	40.5%	0.14 [0.06, 0.34]
Azevedo 2022	0	21	0	42		Not estimable
**Subtotal (95% CI)**		**475**		**762**	**100.0%**	**0.25 [0.05, 1.30]**
Total events	13		88			
Heterogeneity: Tau² = 1.51; Chi² = 10.03, df = 2 (P = 0.007); I² = 80%						
Test for overall effect: Z = 1.64 (P = 0.10)						

Test for subgroup differences: Chi² = 3.37, df = 1 (P = 0.07), I² = 70.3%

Risk Ratio
M-H, Random, 95% CI

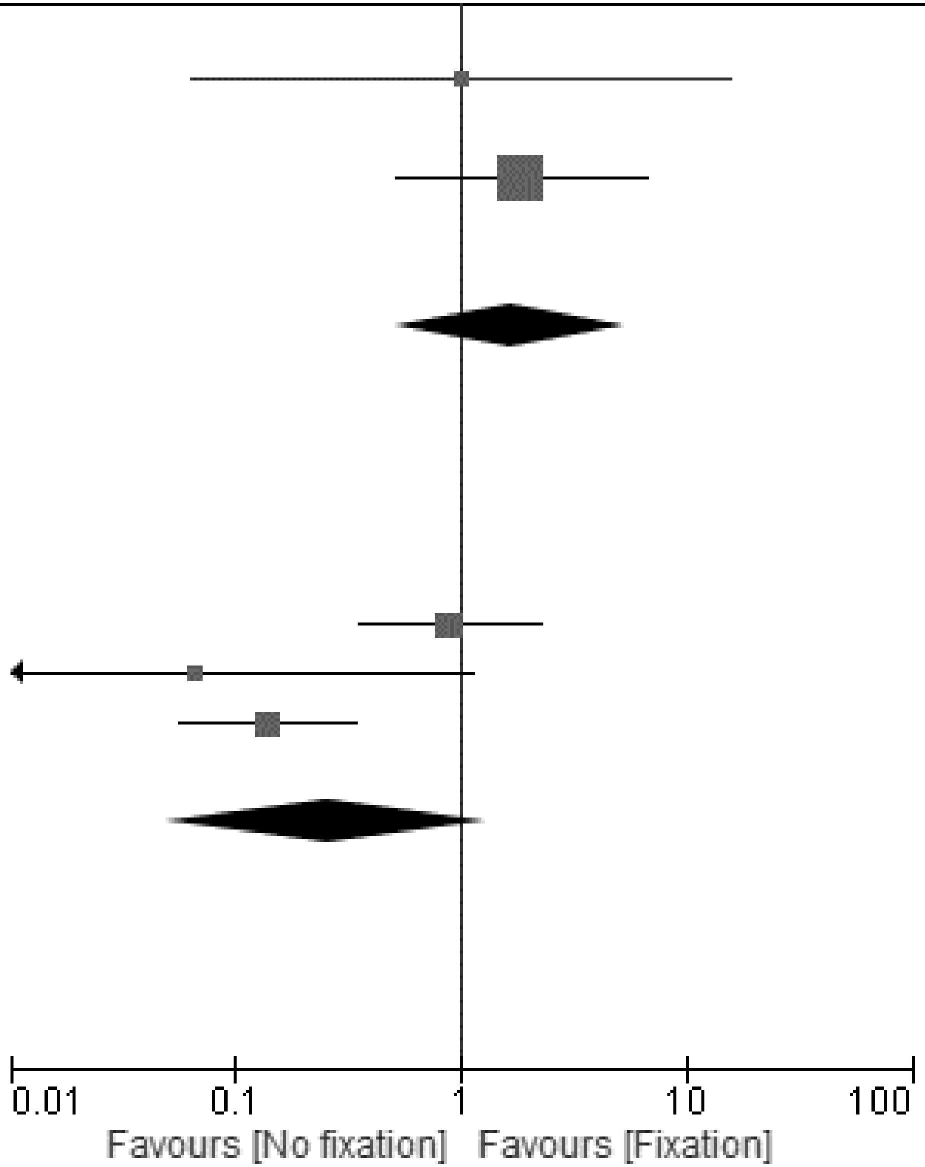

Supplementary Fig. 2B

Study or Subgroup	No fixation Events	No fixation Total	Fixation Events	Fixation Total	Weight	Risk Ratio M-H, Random, 95% CI
**3.5.1 Tacker**						
Moreno-Egea 2004	1	85	1	85	50.7%	1.00 [0.06, 15.73]
Parshad 2005	0	25	0	25		Not estimable
Wang 2018	0	38	7	38	49.3%	0.07 [0.00, 1.13]
Abd-Raboh 2018	0	27	0	31		Not estimable
**Subtotal (95% CI)**		**175**		**179**	**100.0%**	**0.26 [0.02, 4.22]**
Total events	1		8			
Heterogeneity: Tau² = 1.98; Chi² = 1.98, df = 1 (P = 0.16); I² = 49%						
Test for overall effect: Z = 0.94 (P = 0.35)						
**3.5.2 Staple**						
Koch 2006	5	18	3	20	100.0%	1.85 [0.51, 6.67]
**Subtotal (95% CI)**		**18**		**20**	**100.0%**	**1.85 [0.51, 6.67]**
Total events	5		3			
Heterogeneity: Not applicable						
Test for overall effect: Z = 0.94 (P = 0.35)						
**3.5.3 Glue**						
Shen 2017	0	80	0	80		Not estimable
Habeeb 2020	5	266	72	532	100.0%	0.14 [0.06, 0.34]
Azevedo 2022	0	21	0	42		Not estimable
**Subtotal (95% CI)**		**367**		**654**	**100.0%**	**0.14 [0.06, 0.34]**
Total events	5		72			
Heterogeneity: Not applicable						
Test for overall effect: Z = 4.33 (P < 0.0001)						
**3.5.4 Suture**						
Bittner 2011	8	150	9	150	100.0%	0.89 [0.35, 2.24]
**Subtotal (95% CI)**		**150**		**150**	**100.0%**	**0.89 [0.35, 2.24]**
Total events	8		9			
Heterogeneity: Not applicable						
Test for overall effect: Z = 0.25 (P = 0.80)						

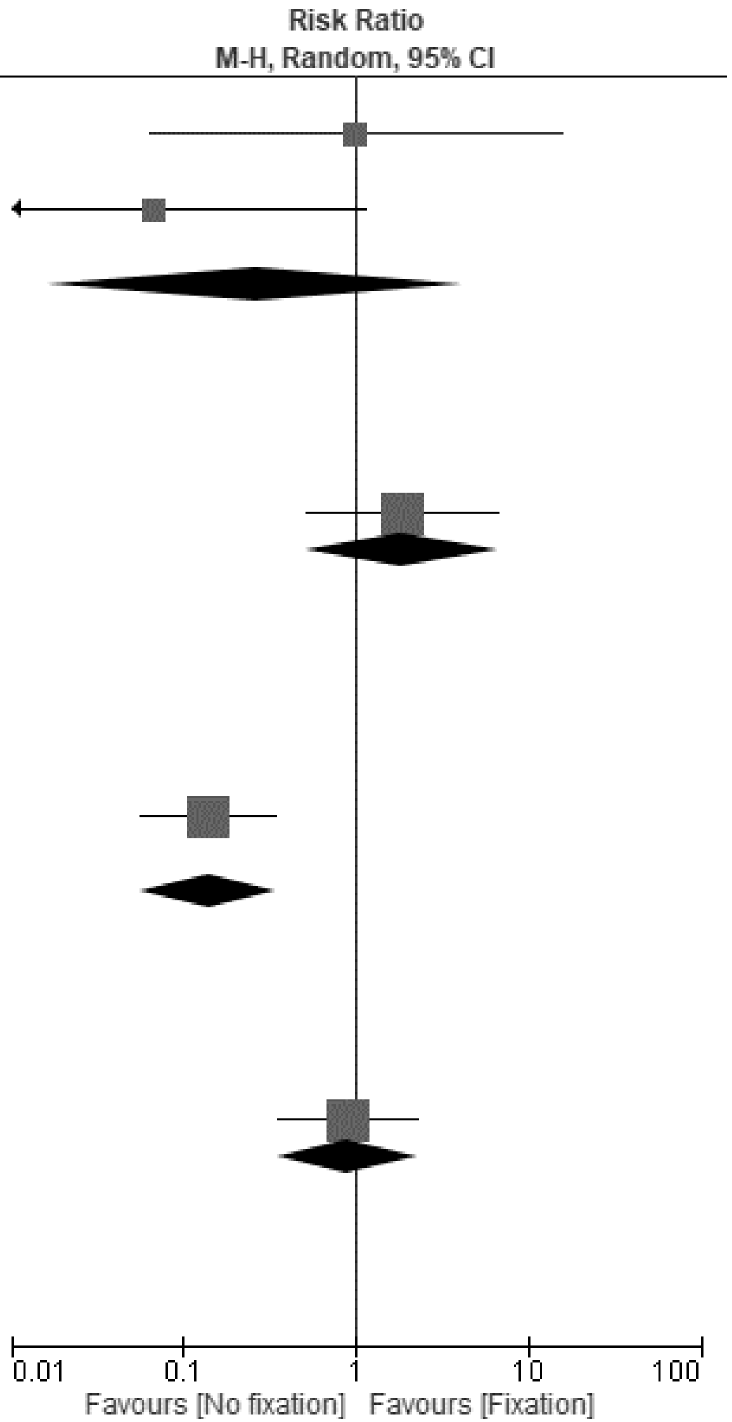

Test for subgroup differences: Chi² = 13.52, df = 3 (P = 0.004), I² = 77.8%

Supplementary Fig. 2C

	No fixation		Fixation			Risk Ratio	Risk Ratio
Study or Subgroup	Events	Total	Events	Total	Weight	M-H, Random, 95% CI	M-H, Random, 95% CI
**3.6.1 Polypropylene**							
Parshad 2005	0	25	0	25		Not estimable	
Bittner 2011	8	150	9	150	40.2%	0.89 [0.35, 2.24]	
Shen 2017	0	80	0	80		Not estimable	
Wang 2018	0	38	7	38	19.4%	0.07 [0.00, 1.13]	
Abd-Raboh 2018	0	27	0	31		Not estimable	
Habeeb 2020	5	266	72	532	40.5%	0.14 [0.06, 0.34]	
**Subtotal (95% CI)**		**586**		**856**	**100.0%**	**0.25 [0.05, 1.30]**	
Total events	13		88				
Heterogeneity: $Tau^2 = 1.51; Chi^2 = 10.03, df = 2 (P = 0.007); I^2 = 80\%$							
Test for overall effect: Z = 1.64 (P = 0.10)							
**3.6.2 3D or self-gripping**							
Moreno-Egea 2004	1	85	1	85	17.8%	1.00 [0.06, 15.73]	
Koch 2006	5	18	3	20	82.2%	1.85 [0.51, 6.67]	
**Subtotal (95% CI)**		**103**		**105**	**100.0%**	**1.66 [0.52, 5.31]**	
Total events	6		4				
Heterogeneity: $Tau^2 = 0.00; Chi^2 = 0.16, df = 1 (P = 0.69); I^2 = 0\%$							
Test for overall effect: Z = 0.85 (P = 0.39)							

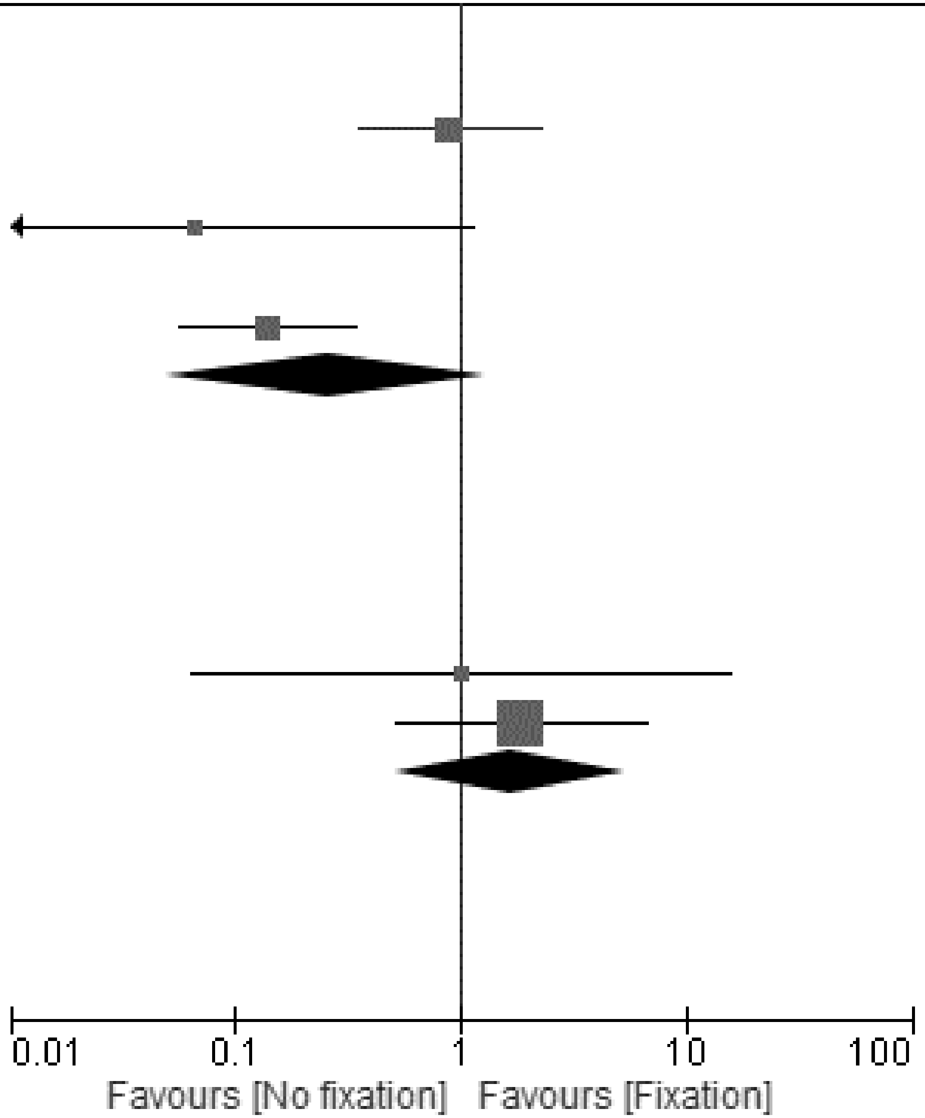

Test for subgroup differences: $Chi^2 = 3.37, df = 1 (P = 0.07), I^2 = 70.3\%$

Supplementary
Fig. 3A

	No fixation			Fixation				Mean Difference
**Study or Subgroup**	**Mean**	**SD**	**Total**	**Mean**	**SD**	**Total**	**Weight**	**IV, Random, 95% CI**
**3.7.1 TEP**								
Ferzli 1999	3.5	1	50	3.5	1	50	33.1%	0.00 [-0.39, 0.39]
Parshad 2005	2.12	1.51	25	2.68	1.63	25	17.5%	-0.56 [-1.43, 0.31]
Garg 2011	7.96	1.15	52	7.77	1.3	48	29.6%	0.19 [-0.29, 0.67]
Yildirim 2022	7	2	50	8	2	50	19.7%	-1.00 [-1.78, -0.22]
**Subtotal (95% CI)**			**177**			**173**	**100.0%**	**-0.24 [-0.71, 0.24]**
Heterogeneity: $Tau^2 = 0.14$; $Chi^2 = 7.76$, df = 3 (P = 0.05); $I^2 = 61\%$								
Test for overall effect: Z = 0.99 (P = 0.32)								
**3.7.2 TAPP**								
Smith 1999	12	1	253	13	1	249	21.5%	-1.00 [-1.17, -0.83]
Li 2007	3.7	1	30	3.9	1.4	30	21.0%	-0.20 [-0.82, 0.42]
Kalidarei 2019	6.9	1.92	39	8.79	2.27	41	20.5%	-1.89 [-2.81, -0.97]
Khalil 2019	7	2	15	8	2	15	19.2%	-1.00 [-2.43, 0.43]
Meshkati Yazd 2023	6	3	50	18	6	50	17.8%	-12.00 [-13.86, -10.14]
**Subtotal (95% CI)**			**387**			**385**	**100.0%**	**-2.97 [-4.87, -1.08]**
Heterogeneity: $Tau^2 = 4.34$; $Chi^2 = 144.06$, df = 4 (P < 0.00001); $I^2 = 97\%$								
Test for overall effect: Z = 3.08 (P = 0.002)								

Test for subgroup differences: $Chi^2 = 7.53$, df = 1 (P = 0.006), $I^2 = 86.7\%$

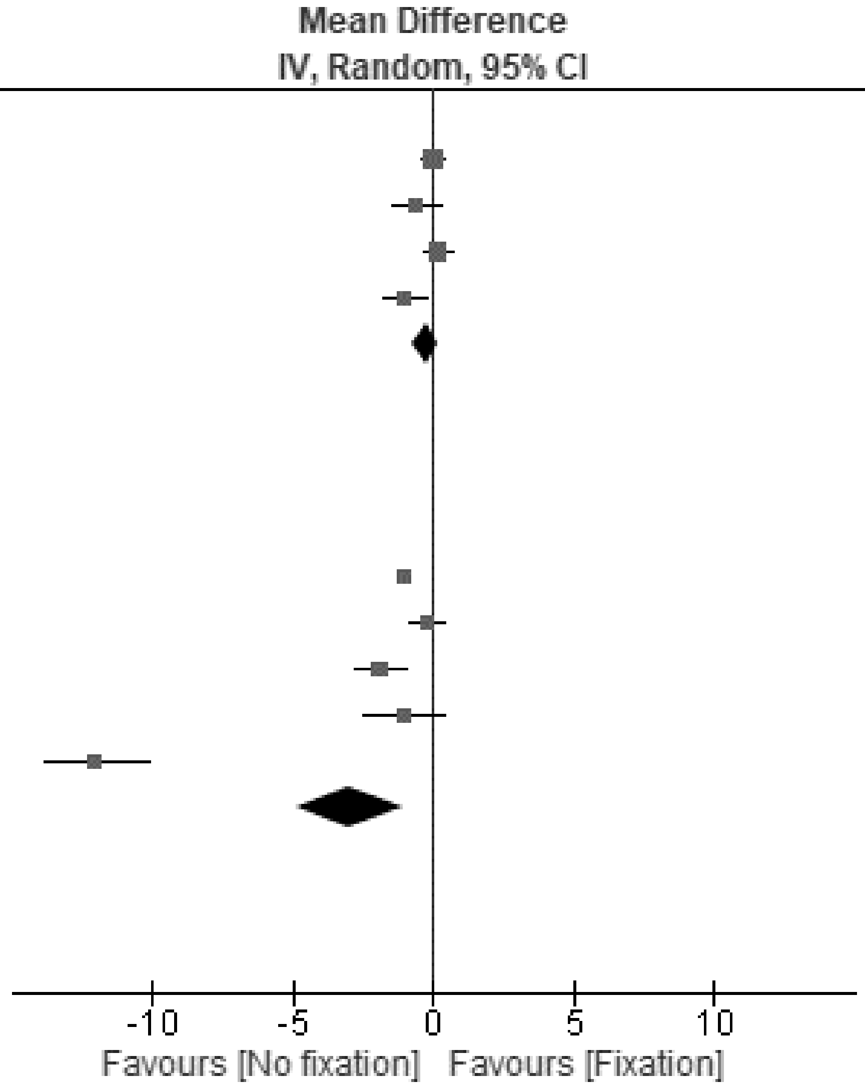

Supplementary Fig. 3B

Study or Subgroup	No fixation Mean	No fixation SD	No fixation Total	Fixation Mean	Fixation SD	Fixation Total	Weight	Mean Difference IV, Random, 95% CI
**3.8.1 General anesthesia**								
Ferzli 1999	3.5	1	50	3.5	1	50	17.6%	0.00 [-0.39, 0.39]
Li 2007	3.7	1	30	3.9	1.4	30	17.4%	-0.20 [-0.82, 0.42]
Kalidarei 2019	6.9	1.92	39	8.79	2.27	41	17.0%	-1.89 [-2.81, -0.97]
Khalil 2019	7	2	15	8	2	15	16.0%	-1.00 [-2.43, 0.43]
Yildirim 2022	7	2	50	8	2	50	17.2%	-1.00 [-1.78, -0.22]
Meshkati Yazd 2023	6	3	50	18	6	50	15.0%	-12.00 [-13.86, -10.14]
**Subtotal (95% CI)**			**234**			**236**	**100.0%**	**-2.48 [-4.30, -0.66]**
Heterogeneity: $Tau^2 = 4.85$; $Chi^2 = 163.73$, df = 5 ($P < 0.00001$); $I^2 = 97\%$								
Test for overall effect: Z = 2.68 (P = 0.007)								
**3.8.2 Spinal anesthesia**								
Garg 2011	7.96	1.15	52	7.77	1.3	48	100.0%	0.19 [-0.29, 0.67]
**Subtotal (95% CI)**			**52**			**48**	**100.0%**	**0.19 [-0.29, 0.67]**
Heterogeneity: Not applicable								
Test for overall effect: Z = 0.77 (P = 0.44)								

Test for subgroup differences: $Chi^2 = 7.75$, df = 1 ($P = 0.005$), $I^2 = 87.1\%$

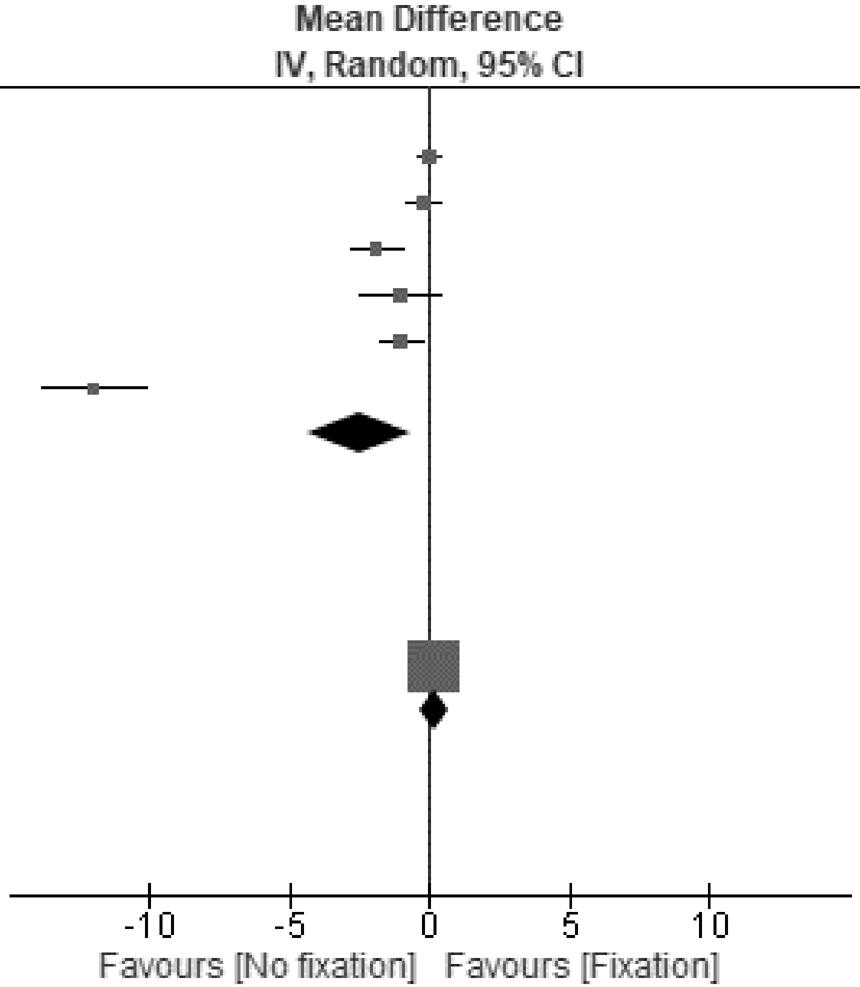

Supplementary Fig. 3C

	No fixation			Fixation				Mean Difference
Study or Subgroup	Mean	SD	Total	Mean	SD	Total	Weight	IV, Random, 95% CI
**3.9.1 Tacker**								
Garg 2011	7.96	1.15	52	7.77	1.3	48	25.8%	0.19 [-0.29, 0.67]
Khalil 2019	7	2	15	8	2	15	24.7%	-1.00 [-2.43, 0.43]
Yildirim 2022	7	2	50	8	2	50	25.5%	-1.00 [-1.78, -0.22]
Meshkati Yazd 2023	6	3	50	18	6	50	24.0%	-12.00 [-13.86, -10.14]
**Subtotal (95% CI)**			**167**			**163**	**100.0%**	**-3.33 [-6.69, 0.03]**
Heterogeneity: Tau² = 11.35; Chi² = 155.67, df = 3 (P < 0.00001); I² = 98%								
Test for overall effect: Z = 1.94 (P = 0.05)								
**3.9.2 Staple**								
Smith 1999	12	1	253	13	1	249	29.9%	-1.00 [-1.17, -0.83]
Ferzli 1999	3.5	1	50	3.5	1	50	27.3%	0.00 [-0.39, 0.39]
Parshad 2005	2.12	1.51	25	2.68	1.63	25	19.2%	-0.56 [-1.43, 0.31]
Li 2007	3.7	1	30	3.9	1.4	30	23.6%	-0.20 [-0.82, 0.42]
**Subtotal (95% CI)**			**358**			**354**	**100.0%**	**-0.45 [-1.08, 0.17]**
Heterogeneity: Tau² = 0.33; Chi² = 24.93, df = 3 (P < 0.0001); I² = 88%								
Test for overall effect: Z = 1.42 (P = 0.15)								

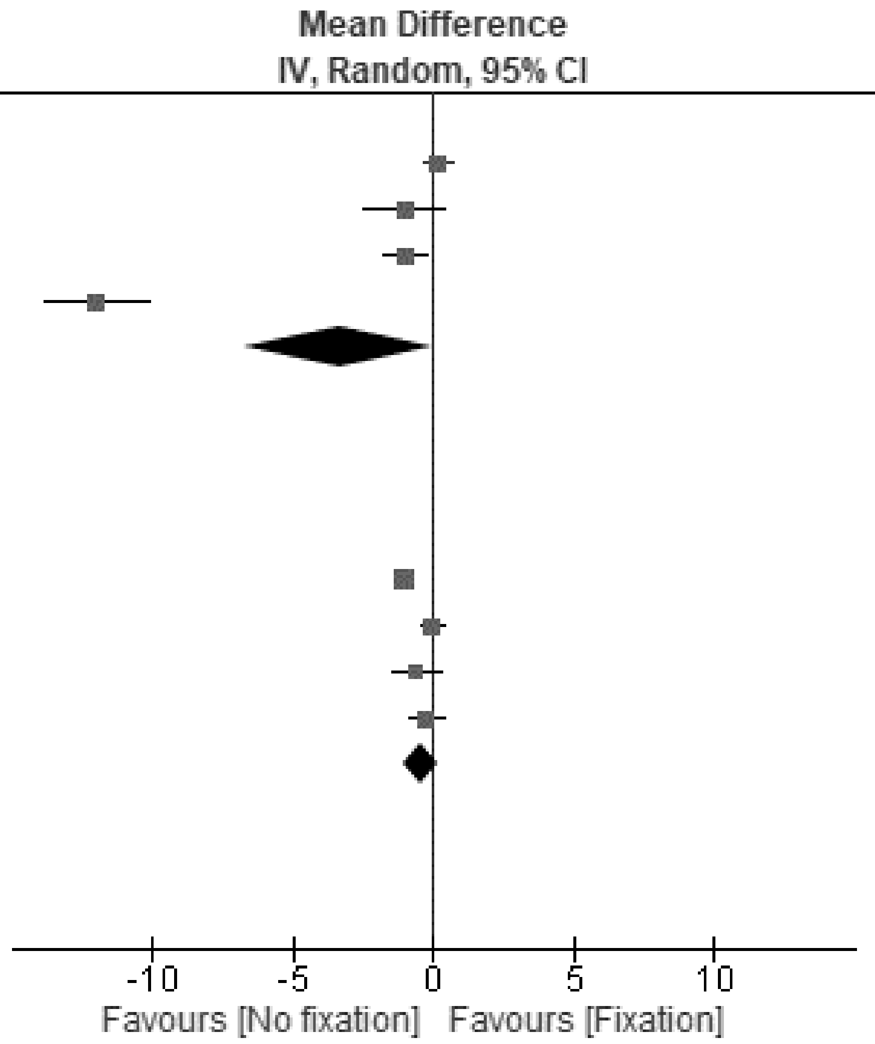

Test for subgroup differences: Chi² = 2.73, df = 1 (P = 0.10), I² = 63.3%

Supplementary Fig. 3D

Study or Subgroup	No fixation Mean	No fixation SD	No fixation Total	Fixation Mean	Fixation SD	Fixation Total	Weight	Mean Difference IV, Random, 95% CI
**3.10.1 Polypropylene**								
Smith 1999	12	1	253	13	1	249	15.7%	-1.00 [-1.17, -0.83]
Ferzli 1999	3.5	1	50	3.5	1	50	15.4%	0.00 [-0.39, 0.39]
Parshad 2005	2.12	1.51	25	2.68	1.63	25	14.3%	-0.56 [-1.43, 0.31]
Garg 2011	7.96	1.15	52	7.77	1.3	48	15.3%	0.19 [-0.29, 0.67]
Kalidarei 2019	6.9	1.92	39	8.79	2.27	41	14.1%	-1.89 [-2.81, -0.97]
Yildirim 2022	7	2	50	8	2	50	14.5%	-1.00 [-1.78, -0.22]
Meshkati Yazd 2023	6	3	50	18	6	50	10.7%	-12.00 [-13.86, -10.14]
**Subtotal (95% CI)**			**519**			**513**	**100.0%**	**-1.90 [-2.97, -0.83]**
Heterogeneity: Tau² = 1.90; Chi² = 182.29, df = 6 (P < 0.00001); I² = 97%								
Test for overall effect: Z = 3.48 (P = 0.0005)								
**3.10.2 3D or self-gripping**								
Li 2007	3.7	1	30	3.9	1.4	30	84.0%	-0.20 [-0.82, 0.42]
Khalil 2019	7	2	15	8	2	15	16.0%	-1.00 [-2.43, 0.43]
**Subtotal (95% CI)**			**45**			**45**	**100.0%**	**-0.33 [-0.90, 0.25]**
Heterogeneity: Tau² = 0.00; Chi² = 1.01, df = 1 (P = 0.31); I² = 1%								
Test for overall effect: Z = 1.12 (P = 0.26)								

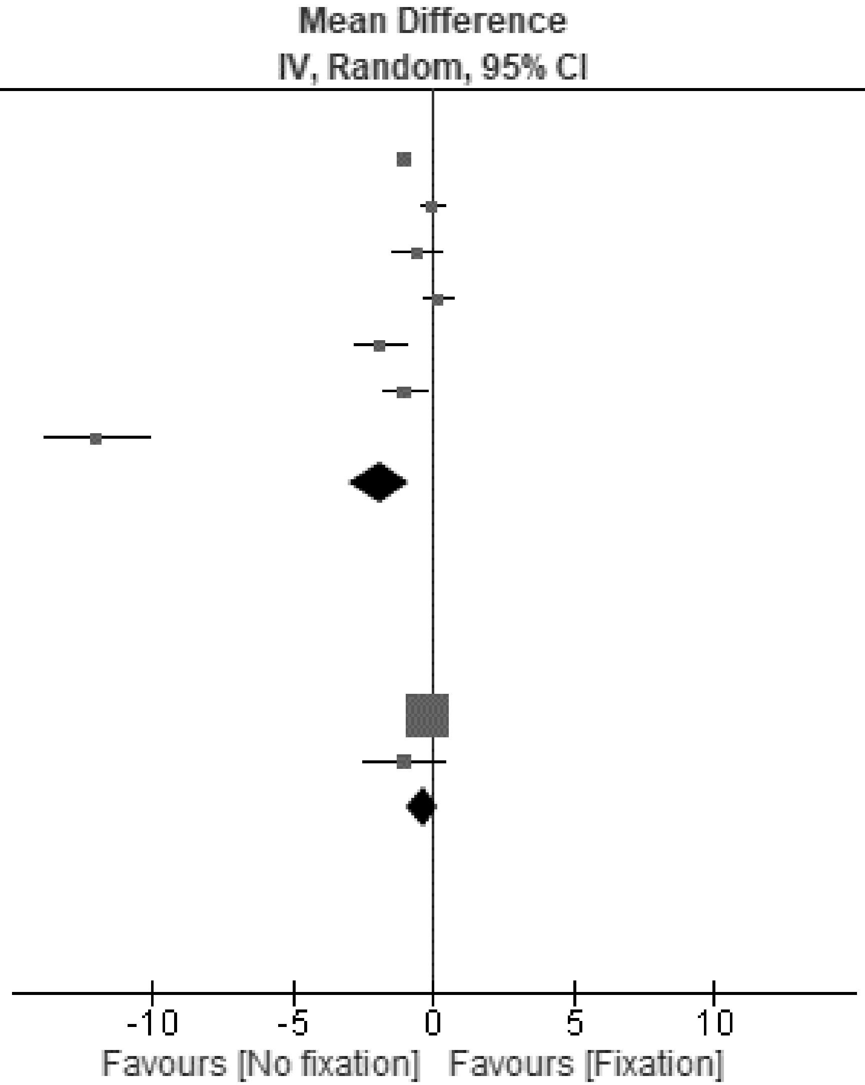

Test for subgroup differences: Chi² = 6.44, df = 1 (P = 0.01), I² = 84.5%

Supplementary Fig. 4A

Study or Subgroup	No fixation Events	No fixation Total	Fixation Events	Fixation Total	Weight	Risk Ratio M-H, Random, 95% CI
Ferzli 1999	0	98	0	98		Not estimable
Moreno-Egea 2004	3	85	0	85	11.2%	7.00 [0.37, 133.48]
Ayyaz 2015	1	31	0	32	9.7%	3.09 [0.13, 73.17]
Claus 2016	0	50	0	10		Not estimable
Shen 2017	4	80	0	80	11.6%	9.00 [0.49, 164.46]
Li 2017	0	50	0	50		Not estimable
Abd-Raboh 2018	0	35	1	35	9.7%	0.33 [0.01, 7.91]
Khalil 2019	0	15	0	15		Not estimable
Habeeb 2020	2	266	3	532	30.7%	1.33 [0.22, 7.93]
Yildirim 2022	2	50	1	50	17.4%	2.00 [0.19, 21.36]
Meshkati Yazd 2023	0	50	1	50	9.7%	0.33 [0.01, 7.99]
**Total (95% CI)**		**810**		**1037**	**100.0%**	**1.78 [0.66, 4.79]**
Total events	12		6			

Heterogeneity: Tau² = 0.00; Chi² = 4.46, df = 6 (P = 0.61); I² = 0%

Test for overall effect: Z = 1.15 (P = 0.25)

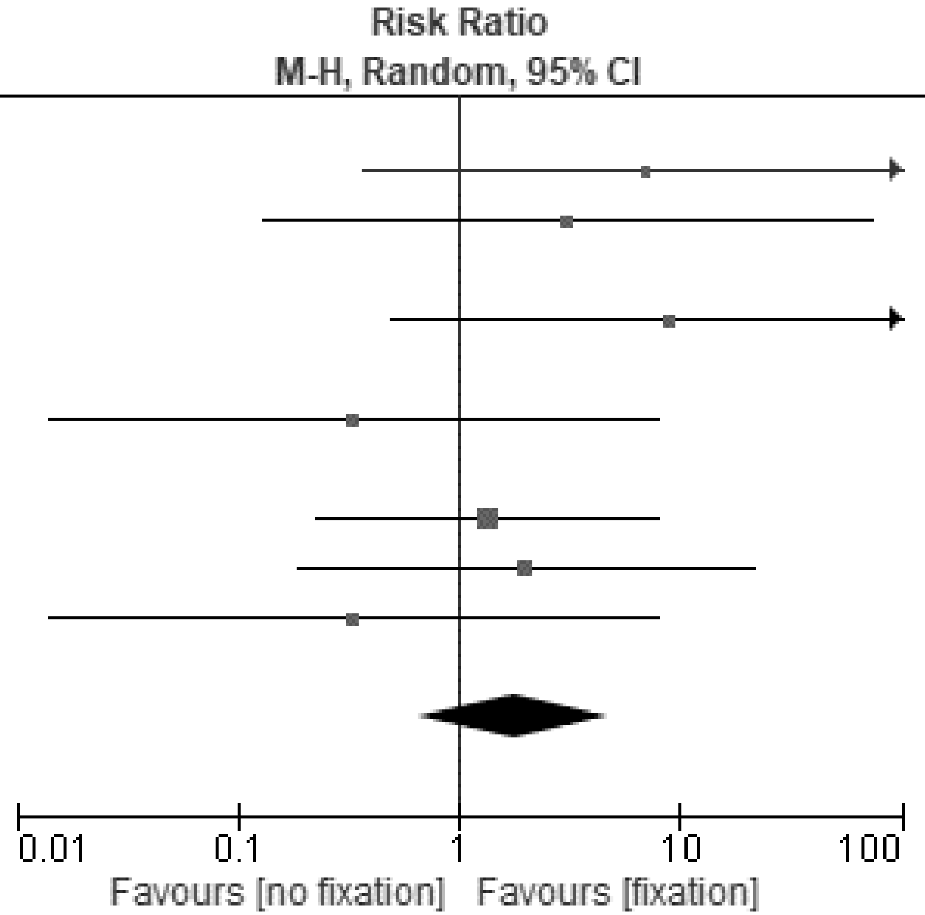

Supplementary Fig. 4B

	No fixation		Fixation			Risk Ratio
Study or Subgroup	Events	Total	Events	Total	Weight	M-H, Random, 95% CI
Moreno-Egea 2004	1	85	1	85	19.2%	1.00 [0.06, 15.73]
Bittner 2011	8	150	9	150	40.2%	0.89 [0.35, 2.24]
Shen 2017	0	80	0	80		Not estimable
Abd-Raboh 2018	0	27	0	31		Not estimable
Habeeb 2020	5	266	72	532	40.6%	0.14 [0.06, 0.34]
**Total (95% CI)**		**608**		**878**	**100.0%**	**0.43 [0.09, 2.06]**
Total events	14		82			

Heterogeneity: Tau² = 1.37; Chi² = 9.36, df = 2 (P = 0.009); I² = 79%

Test for overall effect: Z = 1.06 (P = 0.29)

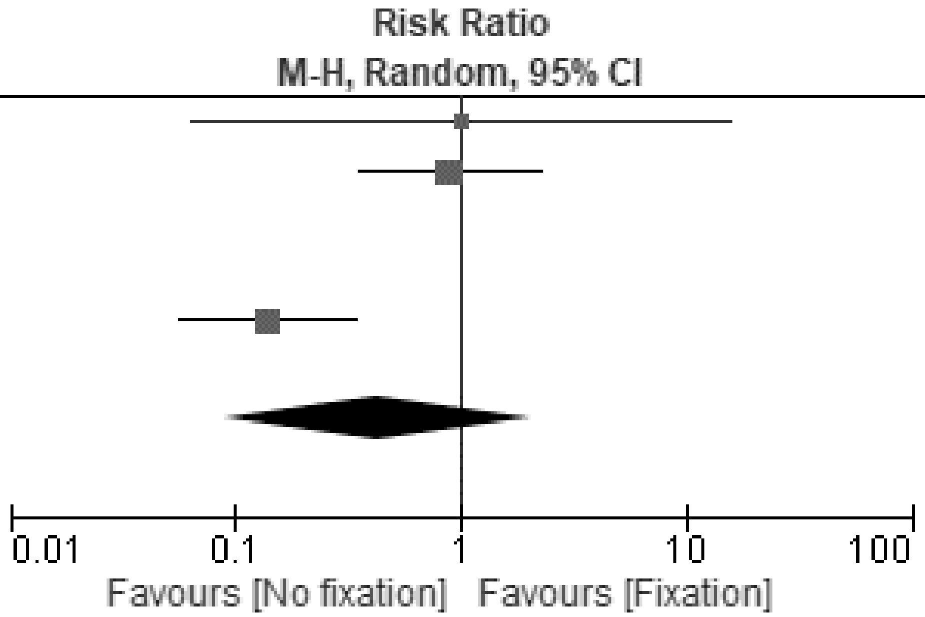

Supplementary Fig. 4C

	No fixation			Fixation				Mean Difference
Study or Subgroup	Mean	SD	Total	Mean	SD	Total	Weight	IV, Random, 95% CI
Ferzli 1999	3.5	1	50	3.5	1	50	25.9%	0.00 [-0.39, 0.39]
Khalil 2019	7	2	15	8	2	15	24.7%	-1.00 [-2.43, 0.43]
Yildirim 2022	7	2	50	8	2	50	25.6%	-1.00 [-1.78, -0.22]
Meshkati Yazd 2023	6	3	50	18	6	50	23.9%	-12.00 [-13.86, -10.14]
**Total (95% CI)**			**165**			**165**	**100.0%**	**-3.37 [-6.58, -0.16]**

Heterogeneity: Tau² = 10.32; Chi² = 154.68, df = 3 (P < 0.00001); I² = 98%
Test for overall effect: Z = 2.06 (P = 0.04)

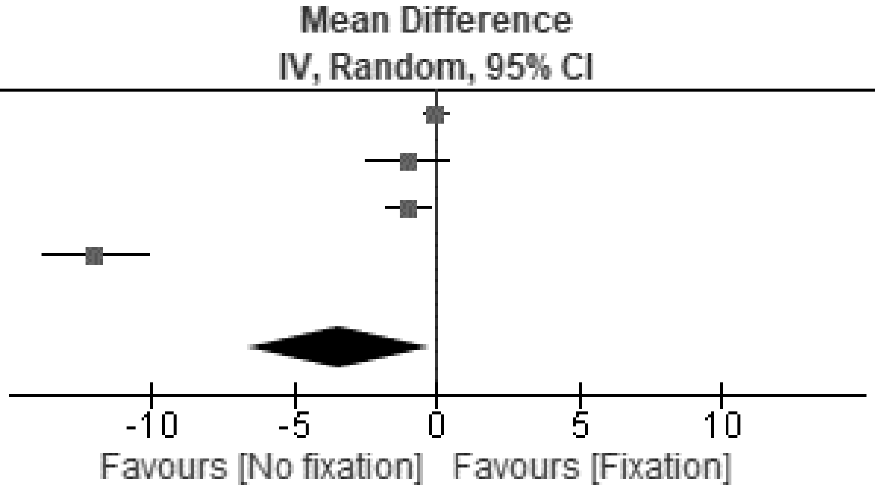

Supplementary Fig. 5

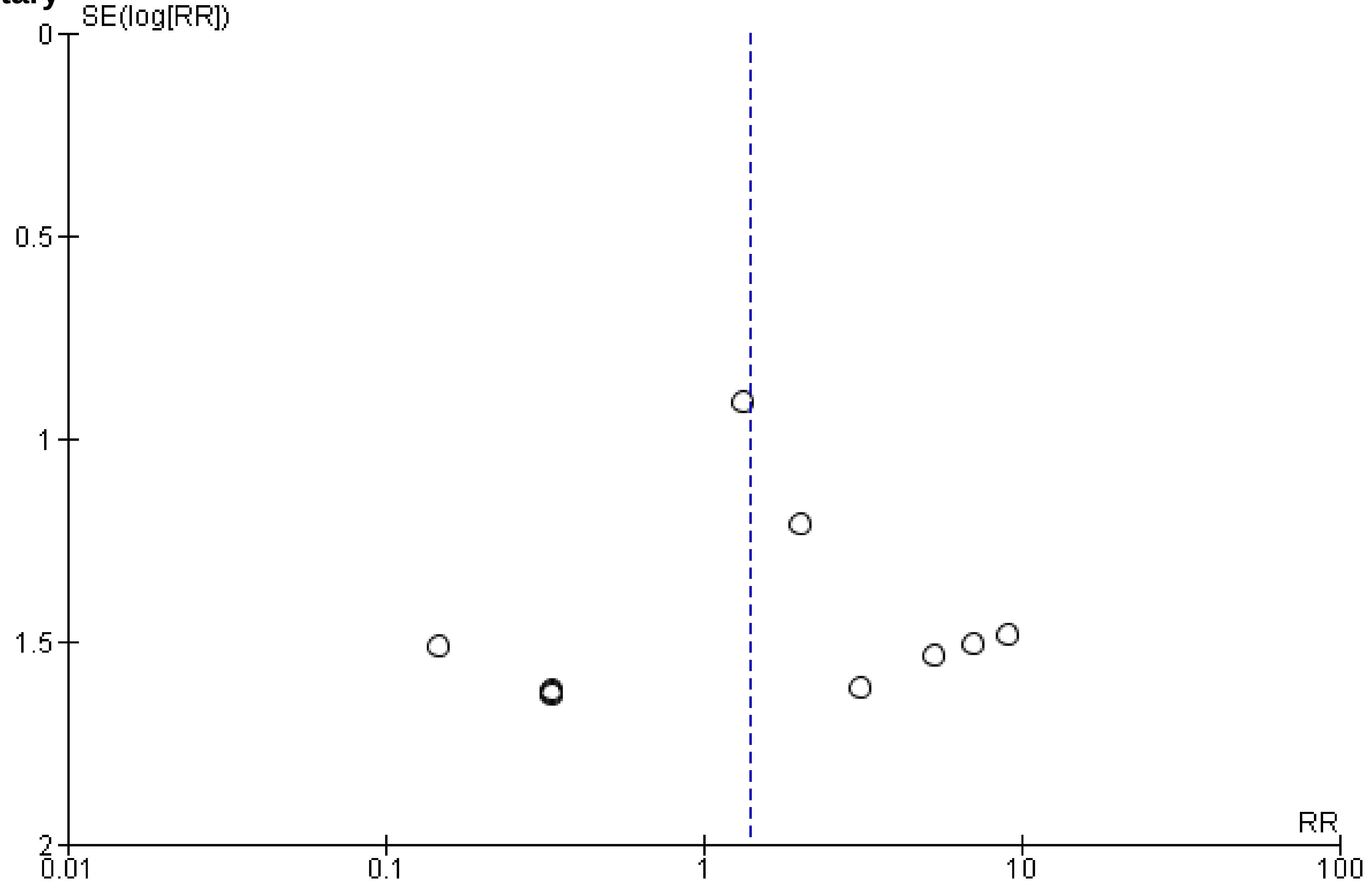

Supplement: Supplementary file 2 — Supplementary file2 (PDF 280 KB) [file 10029_2023_2919_MOESM2_ESM.pdf]
